# Cognitive and physiological responses to prolonged video viewing: Activities of the autonomic nervous system and hypothalamo-pituitary-adrenal axis in relation to video interest

**DOI:** 10.1371/journal.pone.0362672

**Published:** 2026-11-12

**Authors:** Hiroki Matsui, Rina Tanabe, Rikako Mogi, Yuka Sugiura, Hana Yokomachi, Tomoyuki Yokoyama

**Affiliations:** Department of Laboratory Sciences, Gunma University Graduate School of Health Sciences, Maebashi, Japan; Niigata University, JAPAN

## Abstract

In recent years, online lectures and conferences have become widespread, but prolonged video viewing has been suggested to affect psychological state and physiological regulation. However, few studies have objectively examined how such viewing influences autonomic and endocrine activity. This study aimed to evaluate changes in autonomic nervous system (ANS) activity and hypothalamo-pituitary-adrenal (HPA) axis activity during the viewing of high- and low-interest videos. Twenty-six university students (10 males, 16 females; aged 21–24 years) watched high-interest (comedy) and low-interest (lecture) videos (90 minutes each). After viewing each video, they recorded subjective ratings of concentration, comprehension, interest, and fatigue. To assess autonomic nervous system activity, heart rate (HR) and heart rate variability (HRV) analyses, including the low-frequency (LF)/high-frequency (HF) ratio (LF/HF), LF and HF power, and very low-frequency (VLF) power, were performed. As stress markers, saliva samples were collected before, during, and after video viewing to measure cortisol, amylase, and chromogranin A (CgA). Subjective ratings showed that scores for concentration, comprehension, and interest were significantly higher, and fatigue was lower during the high-interest video. Regarding physiological indicators, significant main effects of time were observed for heart rate, LF/HF, LF, HF, and VLF, with overall patterns of lower heart rate and LF/HF and higher LF, HF, and VLF power during viewing. Salivary cortisol also decreased over time during both videos, demonstrating a significant main effect of time. However, no significant changes were observed in salivary amylase or chromogranin A. Furthermore, a significant interest × time interaction was observed for HR, but not for the other physiological indicators. A significant main effect of interest was observed only for LF. These findings indicate that the video conditions differed in subjective cognitive evaluation, with condition-dependent temporal changes detected for HR, whereas no significant interest × time interactions were detected for the other physiological indicators.

## Introduction

The spread of SARS-CoV-2 has led to a rapid increase in remote work and learning. These environmental changes have led to health problems such as adverse effects on sleep, stress, depression, and adjustment disorders [1,2]. In particular, sleep problems have been reported among adults working remotely during the COVID-19 pandemic [3]. In addition, remote learning using devices has become widespread during the COVID-19 pandemic, with some cases exceeding 5 hours per day. Results from a cohort study indicated that screen time and smartphone use were positively correlated with academic stress scores and were associated with increased mental health problems [4,5]. However, the effects of prolonged video viewing on physiological systems, and how the interest level of video content modulates the autonomic nervous system (ANS) and hypothalamo-pituitary-adrenal (HPA) axis activity, remain unclear.

The autonomic nervous system and sympathetic and parasympathetic nervous systems are involved in fatigue- and stress-induced changes in the body. Cardiac autonomic modulation can be assessed indirectly and noninvasively by spectral analysis of heart rate variability, which comprises very low-frequency (VLF), low-frequency (LF), and high-frequency (HF) components. The high-frequency component (HF) is associated with cardiac vagal modulation but is also influenced by respiration and prevailing heart rate. The low-frequency to high-frequency power ratio (LF/HF) has traditionally been used to describe sympathovagal balance, although its validity for this purpose remains controversial and it should not be interpreted as a direct measure of sympathetic activity [6,7]. The VLF may reflect a slow recovery from mental stress and humoral regulation [8]. Increases in LF power have been reported in patients with panic disorder [9], whereas decreased HF indices have been observed in depressive disorders, potentially reflecting reduced cardiac parasympathetic modulation [9,10], indicating that autonomic activity indices are associated with psychiatric disorders, although these indices should not be interpreted as direct measures of autonomic nerve activity. Altered cardiorespiratory coupling has also been reported in young male adults with excessive online gaming [11]. Acute sleep deprivation has also been associated with altered cardiovascular autonomic modulation [12].

Physiological responses to external stimuli are mediated by two primary pathways: the HPA axis and the sympathetic-adrenomedullary (SAM) system [13]. Salivary cortisol is secreted via the HPA axis and enters saliva from plasma as a free hormone, enabling non-invasive monitoring of HPA activity [14]. While cortisol is a well-established biomarker of endocrine responses [15,16], its levels reflect extensive HPA axis regulation across various mental states. Concurrently, the SAM system and autonomic nervous system (ANS) modulate salivary gland function. Salivary amylase, which is regulated by autonomic nerves, and its secretion serves as an indicator of SAM activation [17–19]. Similarly, chromogranin A (CgA) is produced in response to ANS stimulation and is utilized as a sensitive marker for the SAM system [20–22]. Because these systems interact across different time courses, simultaneous evaluation of multiple markers is necessary to comprehensively capture the subject’s physiological state from acute stress to mental engagement or relaxation [23].

However, the effects of prolonged screen viewing on physiological regulation and whether it induces changes in stress or relaxation remain unclear. Most existing studies have focused on smartphones or tablets, which involve close viewing distance and posture-related loads that differ from those of large-screen, offline video viewing. Because the present study used a fixed monitor under standardized conditions, physiological responses were assessed under consistent viewing conditions; however, these responses cannot be attributed solely to video content. This study investigates ANS and HPA axis activity during prolonged viewing of high-interest versus low-interest videos. We aim to clarify whether personal interest level influences physiological responses, including potential shifts toward either arousal or relaxation during prolonged screen exposure. Additionally, we conducted an exploratory analysis of sex differences in physiological responses.

## Materials and methods

### Participants and measurement protocol

The study was conducted with 26 university students (10 males and 16 females) aged 21 to 24 years (22.1 ± 0.7 years). Participants were recruited between January 10th and December 31st, 2021. While recruiting the participants, we explained the study outline and obtained voluntary written consent before participation. The participants were instructed to ensure sufficient sleep the night before the study and to limit alcohol consumption and video viewing. Additionally, they were asked to refrain from eating or consuming caffeine 60 minutes before the measurement and avoid brushing their teeth or engaging in strenuous exercise.

The measurement protocol is illustrated in Fig 1. Before watching the video, each participant completed a questionnaire regarding their current and previous medical history, video-viewing habits, and physical condition on the day of the study. The subjects were fitted with electrodes for heart rate variability measurement, started the measurement, and rested for 10 minutes. The participants watched two types of videos for 90 minutes. The two types of videos used were those of high interest (comedy movies) and low interest (lecture videos in a field different from the participant’s major). Each video was selected through discussions with several non-participating students of the same age group under the following conditions: 1. content that would likely divide students of the same age group into high- and low-interest groups when viewing the two videos; 2. videos that could be completed within the same timeslot, and 3. videos that the participants had not yet viewed. Furthermore, the measurements were conducted after confirming that the participants had not seen the videos. The same participants watched the high- and low-interest videos at the same time of day on different days at random and were measured in the same manner. The viewing order was randomized across participants, with some participants viewing the high-interest video first and others viewing the low-interest video first. This randomization was intended to minimize potential order and period effects. After viewing each video for 90 minutes, the participants rated their interest, concentration, understanding of the content, and fatigue using a 10-point (1–10) numerical rating scale.

**Fig 1:**
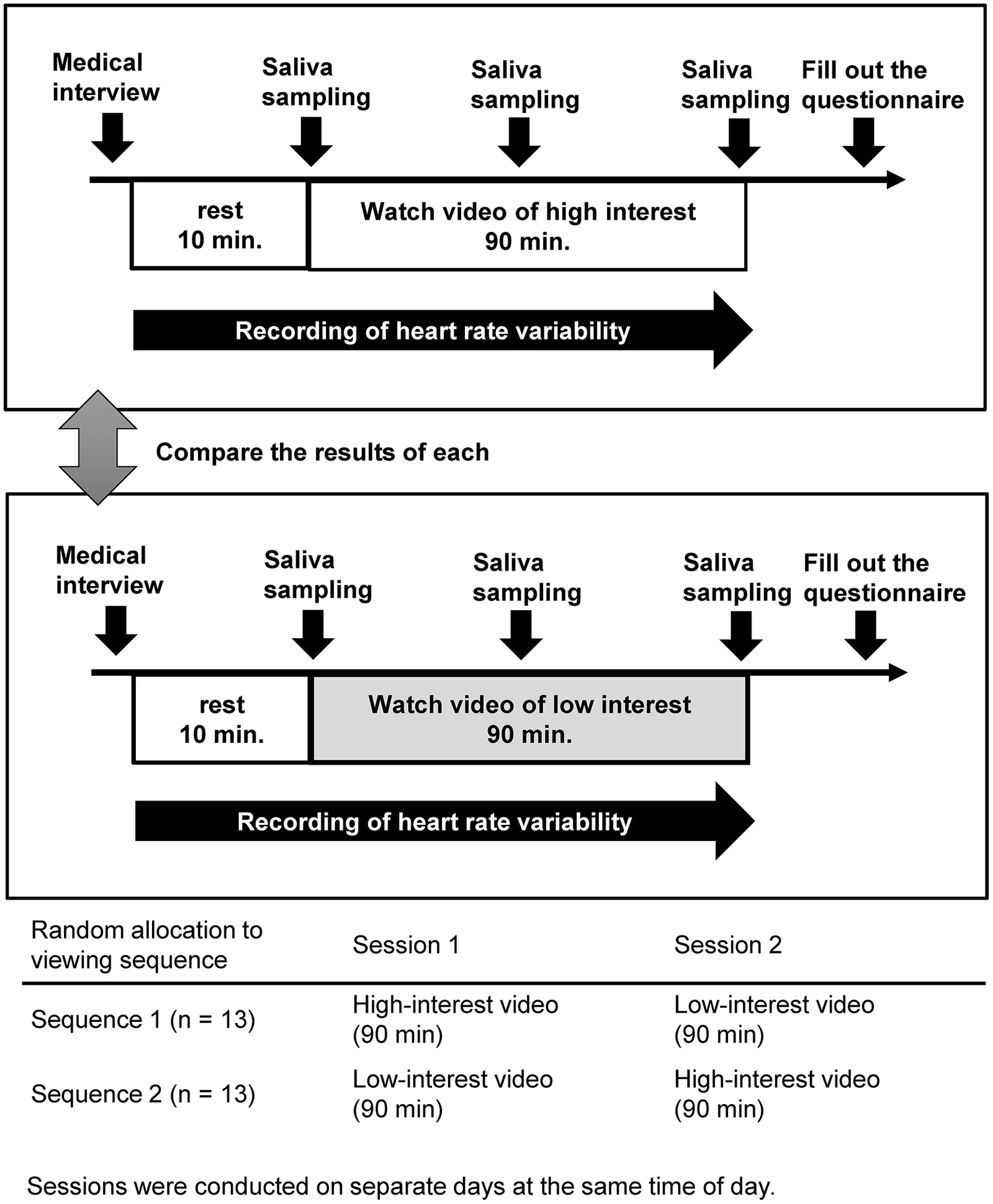
Study design and measurement schedule for viewing high- and low-interest videos. Each participant completed both video conditions in separate sessions conducted on different days at the same time of day. Participants were randomly assigned to one of two viewing sequences: high-interest followed by low-interest, or low-interest followed by high-interest, to minimize potential order and period effects. In each session, participants underwent an interview, rested for 10 minutes, and then watched a 90-minute video. After viewing, they completed a questionnaire about the video. Downward arrows indicate the interview timing, questionnaire responses, and saliva sampling. Rightward arrows indicate the recording periods of heart rate and heart rate variability.

A real-time heart rate variability analyzer (MemCalc/Bonaly Light, GMS, Japan) was used to measure changes in autonomic nervous activity during video viewing. Frequency analysis of the heartbeats obtained from the RR interval was performed using autoregressive power spectrum analysis applied to the time series. Very low-frequency (0.0033-0.04 Hz, Very low-frequency: VLF), low-frequency (0.04-0.15 Hz, Low-Frequency: LF), and high-frequency (0.15-0.4 Hz, High-frequency: HF) spectral bands were identified, and the area under each peak was calculated in absolute units (ms^2^). HF was used as an index of cardiac parasympathetic modulation, and the LF/HF ratio was used as a descriptive frequency-domain HRV measure rather than a direct index of cardiac sympathetic nervous system activity or sympathovagal balance. HF power is influenced by respiration and prevailing heart rate, while LF power reflects multiple regulatory influences, including parasympathetic modulation and baroreflex-related processes. Accordingly, these indices were interpreted cautiously and in conjunction with heart rate and the other physiological measures [6,7]. Time-domain HRV parameters, including RMSSD, were not analyzed because they were not provided by the measurement device used in this study.

Saliva samples were collected at three time points: immediately before watching the video (first time), 45 minutes after the start of the video (second time), and 90 minutes after the start of the video, immediately upon its completion (third time). Saliva collection took place between 09:00 and 12:00 or between 13:00 and 17:00. For each participant, both video-viewing sessions were conducted at the same time of day on separate days, with corresponding saliva samples collected at matched times to minimize time-of-day confounding in comparisons between video conditions. Participants were prohibited from eating or drinking for 15 minutes before saliva collection and were asked to refrain from brushing their teeth or eating. Saliva was collected cryovially by drooling using a Saliva Collection Aid (Salimetrics). The saliva samples were centrifuged (1500 x g) at 4°C for 10 min and stored in a −80°C freezer until analysis.

### Saliva collections and measurements of salivary cortisol concentrations

Cortisol, amylase, and chromogranin A concentrations were measured as stress markers in saliva samples. Salivary cortisol concentrations were measured using the YK241 Cortisol (Saliva) EIA kit (Yanaihara Institute, Shizuoka, Japan), salivary amylase concentrations were measured using the SALIVARY alpha-AMYLASE KINETIC ENZYME ASSAY KIT (SALIMETRICS, PA, USA), and salivary chromogranin A concentrations were measured using the YK070 Human CgA EIA kit (Yanaihara Institute, Shizuoka, Japan) according to the manufacturer’s instructions. Salivary cortisol concentration was measured at 450/620 nm, salivary amylase concentration at 405 nm, and salivary chromogranin A concentration at 490 nm using a BioTek Synergy LX multimode plate reader (Agilent, CA, USA). A standard curve was prepared from the concentration and absorbance results of the standard solutions, and the concentration of each sample was calculated using a regression equation.

### Statistical analysis

The results of each measurement are expressed as median values and corresponding interquartile ranges. The normality of the difference scores (high interest–low interest) was assessed using the Shapiro–Wilk test. When normality was not met, the Wilcoxon signed-rank test was used to compare paired data. The effect size (r) was calculated using the formula r = Z/√N, where *Z* is the standardized test statistic and *N* is the number of observations [24]. To examine temporal changes in HR, HRV indices, and salivary hormones during viewing of two videos, we analyzed the repeated-measures data using a linear mixed-effects model with fixed effects for video interest, time, and their interaction (interest × time).

Time was treated as a categorical variable. Participant-specific random intercepts were included in all models to account for dependence across the two sessions. Additional session-specific random intercepts, nested within participants, were included for HR, LF/HF, HF, cortisol, amylase, and CgA to account for additional within-session dependence. For LF and VLF, these session-specific random intercepts were omitted after initial fits yielded zero session-level variance estimates and estimation warnings. An ARH(1) residual covariance structure was specified within each participant–session combination for HR and the HRV indices, allowing time-specific residual variances and time-dependent correlations. A diagonal residual covariance structure allowing time-specific residual variances was used for the salivary markers. Models were fitted using restricted maximum likelihood, and denominator degrees of freedom were calculated using the Satterthwaite approximation. Model convergence and covariance parameter estimates were checked. Model assumptions were assessed visually using residual histograms, Q–Q plots, residual-versus-fitted plots, and residual distributions by measurement time and video condition. To assess model fit, marginal R^2^ and conditional R^2^ were calculated following the methodology of Nakagawa and Schielzeth [25]. Marginal R^2^ represents the variance explained by all fixed effects collectively, whereas conditional R^2^ represents the variance explained by both fixed and random effects. Model-estimated differences and their 95% confidence intervals were reported to describe the magnitude and precision of between-condition differences averaged across measurement time points and between-condition differences in post-minus-pre viewing changes (S3 Table). Pairwise condition comparisons were Bonferroni-adjusted within each outcome.

Sex differences and interaction effects were tested using a two-way mixed-design ANOVA, with group (high vs. low interest) as a within-subjects factor and sex (men vs. women) as a between-subjects factor. Simple main effects were examined using post hoc comparisons with Bonferroni correction. To examine the relationship between subjective scores and physiological indicators (HRV, salivary hormones), Spearman’s rank correlation coefficient (ρ) was calculated. The analysis assumed no normality, used a two-tailed test, and set the significance level at p = 0.05. All statistical analyses were performed using Prism 9.5.1 (GraphPad Software, Boston, MA, USA) and SPSS Statistics version 31 (IBM Corp., Armonk, NY, USA), and statistical significance was set at p < 0.05.

### Ethics approval and consent to participate

Written informed consent was obtained from all the participants. This study was approved by the Gunma University Medical Ethics Review Committee for Human Subjects (approval number: HS2020-147). All the measurements on the participants were performed in accordance with the Declaration of Helsinki.

## Results

### The subjective score of the participants for each video

The participants’ subjective scores after viewing each video are shown in Fig 2 and Table 1. The Shapiro–Wilk test indicated that the subjective scores after viewing the high-interest and low-interest videos were not normally distributed. The Wilcoxon signed-rank test showed that the scores for Concentration, Comprehension, and Interest were significantly higher after watching the high-interest videos than after watching the low-interest videos. Conversely, the fatigue score after watching the high-interest videos was significantly lower than the score after watching the low-interest videos. Each effect size was large, suggesting that viewing videos of different interests significantly influenced subjects’ subjective scores (Table 1).

**Fig 2:**
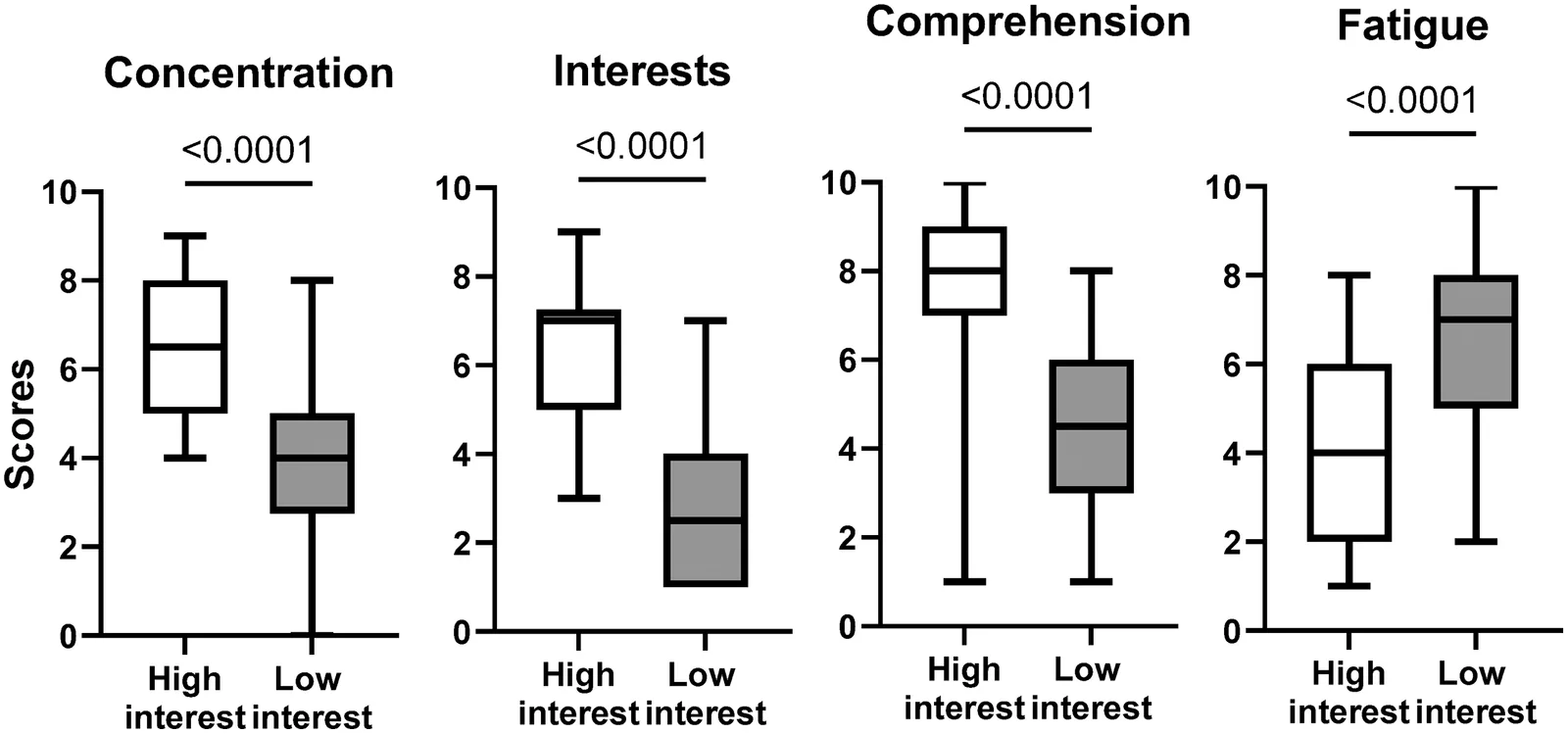
Comparison of numerical rating scores for concentration, comprehension, interest, and fatigue after watching a video. White boxes indicate results after viewing videos of high interest, and gray boxes indicate results after viewing videos of low interest. Significant differences between the two videos were analyzed using the Wilcoxon signed-rank test.

**Table 1:** Comparison of subjective scores for watching high-interest and low-interest videos.

	High-interest	Low-interest	*Z*	P value	r
Concentration	6.50 (5.00–8.00)	4.00 (3.00–5.00)	−3.82	<0.001	−0.75
Interests	7.00 (5.25–7.00)	2.50 (1.25–4.00)	−4.39	<0.001	−0.86
Comprehension	8.00 (7.00–8.75)	4.50 (3.00–6.00)	−3.95	<0.001	−0.78
Fatigue	4.00 (2.25–5.75)	7.00 (5.00–8.00)	3.84	<0.001	0.75

Data are expressed as the median (interquartile range). The normality of difference scores was tested using the Shapiro–Wilk test. Significant differences between high and low interest scores were analyzed using the Wilcoxon matched-pairs signed-rank test. Effect size r = Z/√N.

### Changes in HRV and Salivary Hormones during Video Viewing

Fig 3, Fig 4 and Tables 2, 3 show the results of the time series changes in heart rate, LF/HF, LF, HF, VLF, salivary cortisol, amylase, and CgA concentrations during video viewing, respectively. Using a linear mixed model, we examined the interaction effects of viewing high-interest versus low-interest videos on each metric (Table 3). Table 4 presents model-estimated mean differences between video conditions, averaged across measurement time points, and their 95% confidence intervals. Model-estimated between-condition differences in post-minus-pre viewing changes and their 95% confidence intervals are presented in S3 Table.

**Fig 3:**
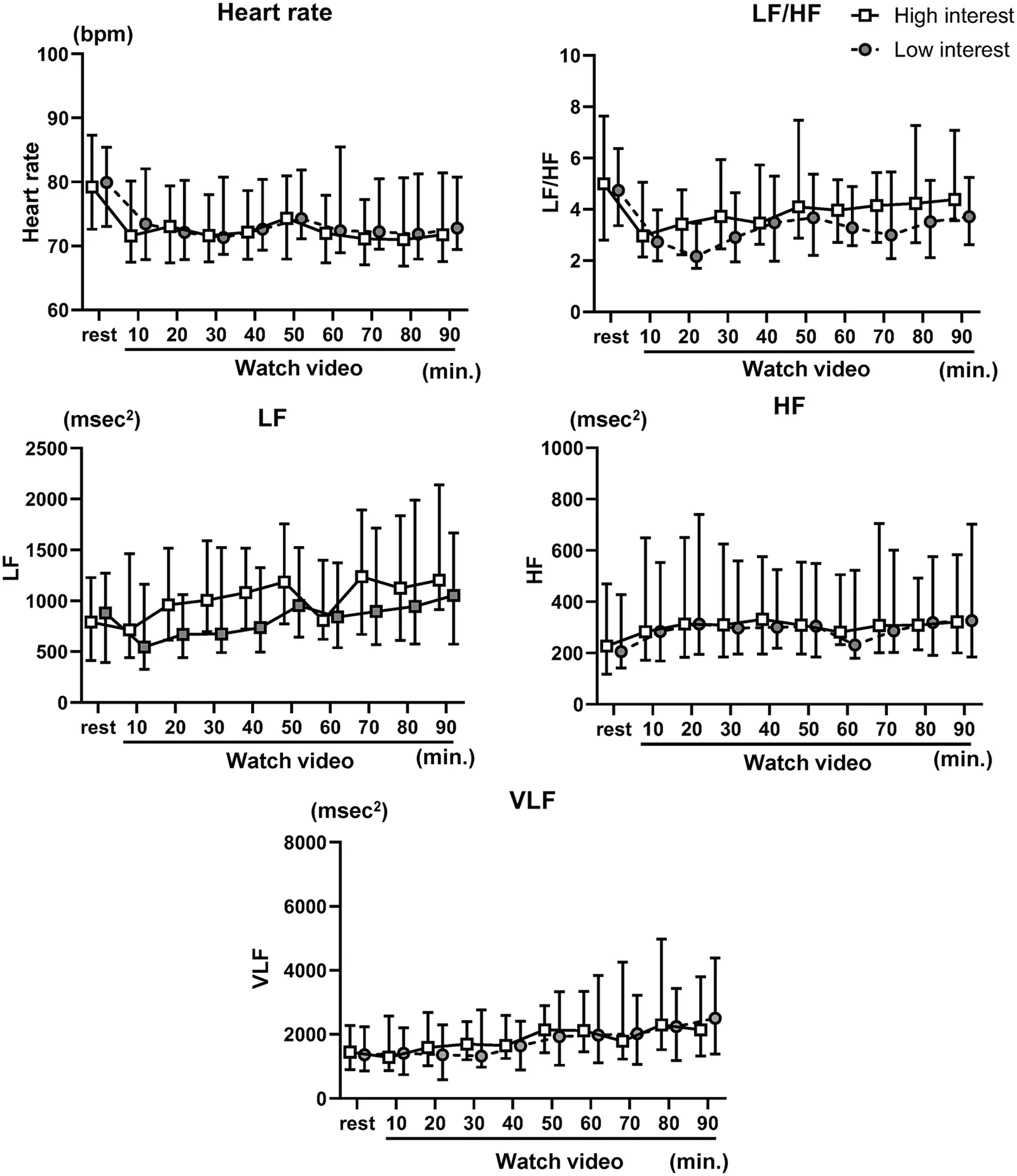
Comparison of heart rate, LF/HF, LF, HF, and VLF results during video viewing. White squares indicate results during video viewing of high interest, and gray circles indicate results during low interest. Data are presented as median values and corresponding interquartile ranges.

**Fig 4:**
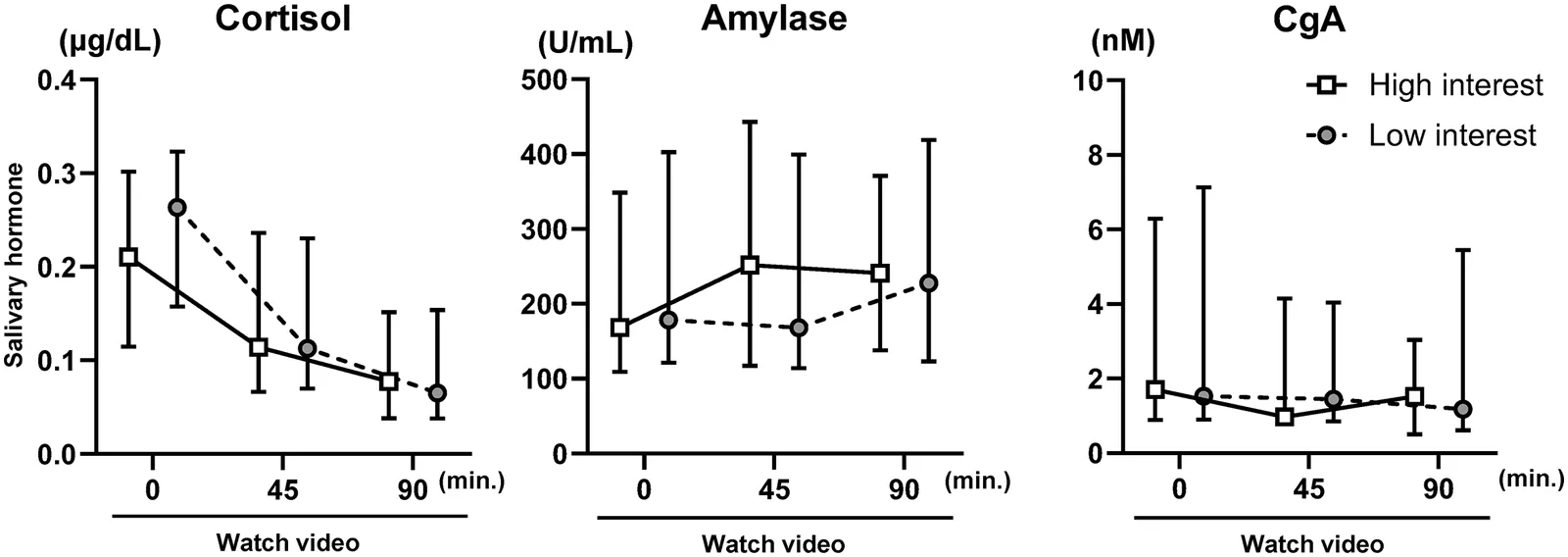
Comparison of salivary cortisol, salivary amylase, and salivary CgA concentrations before, during, and after watching the videos. White squares indicate results of high interest during video viewing, and gray circles indicate results of low interest. Data are presented as median values and corresponding interquartile ranges.

**Table 2:** Heart rate, HRV indicators, and salivary hormone levels during viewing of high-interest and low-interest videos.

	High-interest		Low-interest	
	pre	post	pre	post
Heart rate (beat/min)	79.2 (74.2–84.5)	71.8 (68.5–80.3)	80.7 (74.2–84.9)	74.0 (69.9–79.8)
LF/HF	5.00 (2.92–7.21)	4.38 (3.66–6.81)	4.73 (3.54–6.19)	3.71 (2.74–5.21)
LF (msec^2^)	791.6 (431.1–1147.2)	1203.6 (972.6–2061.6)	880.0 (446.2–1131.8)	1053.1 (597.9–1501.3)
HF (msec^2^)	227.4 (131.0–415.4)	321.1 (214.6–569.5)	205.8 (148.0–421.3)	326.5 (190.8–665.3)
VLF (msec^2^)	1449.5 (986.9–2014.8)	2144.8 (1379.1–3699.8)	1364.3 (901.4–2209.6)	2503.1 (1432.5–4352.2)
Cortisol (µg/dL)	0.211 (0.128–0.295)	0.077 (0.040–0.139)	0.263 (0.164–0.320)	0.065 (0.038–0.145)
Amylase (U/mL)	168.4 (110.9–322.0)	241.1 (142.5–349.2)	178.6 (122.3–381.2)	227.6 (125.7–385.9)
CgA (pmol/mL)	1.71 (0.92–5.06)	1.52 (0.51–2.75)	1.53 (0.93–5.91)	1.18 (0.64–4.07)

Data are expressed as the median (interquartile range).

**Table 3:** Comparison of heart rate, HRV indicators, and salivary hormone levels during viewing of high-interest and low-interest videos.

	df1	df2	F	p	R^2^m	R^2^c
[HR]					0.058	0.933
Interest	1	25.697	2.646	0.116		
Time	9	129.544	20.191	<.001		
interest × time	9	129.544	2.802	0.005		
[LF/HF]					0.055	0.460
Interest	1	41.489	2.005	0.164		
Time	9	84.119	8.348	<.001		
interest × time	9	84.119	1.069	0.394		
[LF]					0.066	0.728
Interest	1	82.513	18.833	<.001		
Time	9	114.110	7.525	<.001		
interest × time	9	114.110	0.927	0.505		
[HF]					0.018	0.826
Interest	1	10.342	0.015	0.906		
Time	9	90.373	3.031	0.003		
interest × time	9	90.373	0.699	0.709		
[VLF]					0.077	0.672
Interest	1	49.062	3.861	0.055		
Time	9	145.097	5.892	<.001		
interest × time	9	145.097	1.095	0.370		
[Cortisol]					0.157	0.819
Interest	1	24.459	0.164	0.689		
Time	2	62.043	37.172	<.001		
interest × time	2	62.043	0.358	0.701		
[amylase]					0.008	0.843
Interest	1	39.008	0.755	0.390		
Time	2	53.911	0.489	0.616		
interest × time	2	53.911	1.840	0.169		
[CgA]					0.004	0.891
Interest	1	24.660	0.661	0.424		
Time	2	51.000	0.244	0.785		
interest × time	2	51.000	1.658	0.201		

Values are from Type III tests of fixed effects in the linear mixed model fitted using restricted maximum likelihood. Denominator degrees of freedom were calculated using the Satterthwaite approximation. Time was treated as a categorical variable. df1 and df2 indicate the numerator and denominator degrees of freedom, respectively. R^2^m and R^2^c indicate marginal R^2^ and conditional R^2^ values for the full model for each outcome.

**Table 4:** Model-estimated mean differences in physiological indicators between high- and low-interest video conditions.

	Estimate Mean difference (High interest – Low interest)	95% CI
Heart rate (beat/min)	−2.175	−4.926 to 0.575
LF/HF	0.594	−0.253 to 1.440
LF (msec^2^)	223.241	120.917 to 325.565
HF (msec^2^)	6.998	−120.779 to 134.775
VLF (msec^2^)	481.103	−10.882 to 973.087
Cortisol (µg/dL)	−0.009	−0.052 to 0.035
Amylase (U/mL)	−30.344	−101.000 to 40.312
CgA (pmol/mL)	−0.746	−2.638 to 1.145

Differences were calculated as high-interest minus low-interest using estimated marginal means from linear mixed models, averaged equally across all measurement time points. Negative values indicate lower estimated values in the high-interest condition. Confidence intervals for pairwise comparisons were Bonferroni-adjusted within each outcome. Estimates are presented on the original measurement scale. CI, confidence interval.

The observed heart rate pattern showed lower values from 10 min after viewing onset through 90 min than before viewing in both conditions. In the linear mixed model, significant main effects of time, F(9, 129.544) = 20.191, p < 0.001, and a significant interest × time interaction, F(9, 129.544) = 2.802, p = 0.005, were observed for heart rate, whereas the main effect of interest was not significant, F(1, 25.697) = 2.646, p = 0.116. Across time points, heart rate was not significantly different between conditions (estimated mean difference = −2.175, 95% CI: −4.926 to 0.575). The post-minus-pre change in heart rate was more negative in the high-interest condition than in the low-interest condition (between-condition difference in change = −3.558, 95% CI: −6.628 to −0.488; S3 Table; unadjusted comparison). Regarding HRV, the linear mixed model showed a significant main effect of time for LF/HF, LF, HF, and VLF [F(9, 84.119) = 8.348, p < 0.001; LF: F(9, 114.110) = 7.525, p < 0.001; HF: F(9, 90.373) = 3.031, p = 0.003; VLF: F(9, 145.097) = 5.892, p < 0.001]. A significant main effect of interest was observed only for LF, but not for LF/HF, HF, or VLF [F(1, 41.489) = 2.005, p = 0.164; LF: F(1, 82.513) = 18.833, p < 0.001; HF: F(1, 10.342) = 0.015, p = 0.906; VLF: F(1, 49.062) = 3.861, p = 0.055]. No significant interest × time interaction was observed for any HRV indicators [F(9, 84.119) = 1.069, p = 0.394; LF: F(9, 114.110) = 0.927, p = 0.505; HF: F(9, 90.373) = 0.699, p = 0.709; VLF: F(9, 145.097) = 1.095, p = 0.370]. Across time points, LF was significantly higher in the high-interest condition than in the low-interest condition [LF: estimated mean difference = 223.241 (ms^2^), 95% CI: 120.917 to 325.565]. The estimated differences in LF/HF and VLF were also positive, but their confidence intervals included zero [LF/HF: estimated mean difference = 0.594, 95% CI: −0.253 to 1.440; VLF: estimated mean difference = 481.103, 95% CI: −10.882 to 973.087].

Regarding salivary hormones, salivary cortisol showed a significant main effect of time, F(2, 62.043) = 37.172, p < 0.001. Post hoc comparisons showed that cortisol concentrations were lower after video viewing than before viewing when averaged across video conditions (estimated mean difference = −0.107, 95% CI: −0.136 to −0.079). However, no significant main effect of interest, F(1, 24.459) = 0.164, p = 0.689, or interest × time interaction, F(2, 62.043) = 0.358, p = 0.701, was observed. No significant main effects of interest or time, and no significant interest × time interactions, were observed for salivary amylase [interest: F(1, 39.008) = 0.755, p = 0.390; time: F(2, 53.911) = 0.489, p = 0.616; interaction: F(2, 53.911) = 1.840, p = 0.169] or salivary CgA [interest: F(1, 24.660) = 0.661, p = 0.424; time: F(2, 51.000) = 0.244, p = 0.785; interaction: F(2, 51.000) = 1.658, p = 0.201].

The Marginal R^2^ values for each linear mixed model ranged from 0.004 to 0.157, while Conditional R^2^ values ranged from 0.460 to 0.933. The explanatory power of models with fixed effects alone was generally low, whereas the overall explanatory power of models including random effects showed variation depending on the metric.

Also, we examined the main effect of sex and the video condition x sex interaction for each indicator. However, neither effect was statistically significant for any of the indicators included in this exploratory analysis (S1 Table).

### Relationship between subjective assessment and various physiological indicators

We examined correlations between subjective scores (concentration, interest, comprehension, fatigue) and physiological indicators (HRV, salivary hormones) (S2 Table). During viewing of low-interest videos, heart rate showed a weak negative correlation with interest (Spearman’s ρ = −0.389, p < 0.05), salivary cortisol levels showed a weak negative correlation with concentration (ρ = −0.423, p < 0.05), and comprehension (ρ = −0.459, p < 0.05). However, no significant correlations were found with other indicators. These results suggest limited correspondence between participants’ subjective scores and objective physiological indicators.

## Discussion

In this study, we examined ANS and the HPA-related responses during viewing high-interest and low-interest videos. Subjective cognitive responses differed clearly between the two video conditions, whereas a significant interest × time interaction was observed for HR but not for the other physiological indicators. HRV indices changed over time during viewing, and salivary cortisol decreased over time under both conditions.

### 1. An interest × time interaction was detected for HR, whereas the other ANS indicators showed no significant interactions

ANS- and SAM-related markers other than HR showed no significant interest × time interaction between high-interest and low-interest viewing conditions. However, average levels across measurement time points differed between conditions only for LF (Table 4). For HR, the significant interaction indicated a difference in temporal trajectories between conditions; the supplementary endpoint contrast also showed a more negative post-minus-pre change in the high-interest condition (S3 Table), although this comparison was not adjusted for multiple comparisons. The main effect of condition on LF should be distinguished from a difference in temporal trajectory, as no significant interest × time interaction was detected for LF. Because these estimates include pre-viewing measurements, the condition main effects do not necessarily represent differences induced by video viewing. These time-related changes cannot be attributed specifically to video viewing, as elapsed time, habituation to the experimental setting, and prolonged seated rest may also have contributed. Salivary amylase and CgA showed no clear time-dependent or interest-dependent changes. In all models, Marginal R^2^ values were generally small, while Conditional R^2^ values varied across outcomes. The increase from marginal to conditional R^2^ indicates that participant-specific and, where included, session-specific random intercepts accounted for additional variability. However, these model-level measures do not quantify the effects of video interest, time, or their interaction separately. In this study, heart rate and LF/HF decreased during both video viewings, while LF, HF and VLF increased over time. Previous studies comparing pleasant, unpleasant, and neutral movies have reported heart rate deceleration during emotional film viewing, which may reflect orienting and attentional processing rather than relaxation alone [26]. Additionally, viewing animal videos has also been reported to reduce HR and blood pressure and attenuate cardiovascular responses to stress compared to human or blank-screen videos [27]. In contrast, tasks requiring sustained cognitive effort or mental arithmetic have been shown to increase HR and sympathetic activity, reflecting cognitive stress [28]. This study observed reductions in heart rate, increases in high-frequency (HF) components, and decreases in the low-frequency/high-frequency (LF/HF) ratio, a pattern that may be consistent with reduced cardiovascular arousal during viewing but does not establish that the videos imposed little cognitive load. The gradual increase in VLF power is thought to reflect progressive humoral regulation and thermoregulation after prolonged exposure, rather than immediate autonomic output [6]. Furthermore, while the LF/HF ratio is widely used, its validity as an index of sympathetic activity or sympathovagal balance remains controversial [6,7]. LF reflects multiple regulatory influences, and changes in LF/HF can arise from changes in LF, HF, or both. Thus, a decrease in LF/HF should not be interpreted directly as reduced sympathetic activity. In the present study, both LF and HF showed significant main effects of time and increased during viewing. The decrease in LF/HF therefore occurred alongside increases in both components, rather than a decrease in LF, and may reflect their relative changes. Across time points, LF was higher in the high-interest condition, whereas HF showed no significant main effect of interest. Although the estimated LF/HF ratio was higher in the high-interest condition, this difference was not statistically significant (Table 4); the relative contributions of LF and HF to the ratio were not directly quantified. Higher LF should not, however, be interpreted as evidence of greater sympathetic activity. Neither LF nor HF showed a significant interest × time interaction, providing no evidence that their temporal trajectories differed between conditions. The decrease in heart rate and increase in HF over time may be consistent with increased cardiac vagal modulation and a more relaxed physiological state; however, HF is also influenced by respiration and prevailing heart rate [6], and these findings do not establish parasympathetic activation or relaxation. The concurrent increase in LF does not necessarily contradict this interpretation, because LF is not a specific marker of sympathetic activity.

SAM-related markers (amylase and CgA) also showed no clear changes dependent on interest in the videos, but these non-significant findings do not establish the absence of sympathetic arousal. Previous research has reported decreased salivary amylase with relaxing visual stimuli [29] and increased amylase with unpleasant affective images [30]. However, the confidence intervals for the condition differences include both positive and negative values (Table 4), and limited measurement sensitivity, sampling timing, or statistical power may also have contributed to the non-significant results. The present measurements did not detect significant changes in amylase or CgA, but this does not establish that emotional arousal was limited.

### 2. Cortisol levels decreased during video viewing irrespective of interest in the video

A key finding of this study was a significant decrease in salivary cortisol over time across the two viewing conditions. As cortisol is the major marker of the HPA axis, this decrease may be consistent with reduced HPA axis activity during the 90-minute period, but does not by itself establish a reduction in physiological stress attributable to video viewing. While aggressive or highly arousing visual content was associated with elevated cortisol [31], mild cognitive engagement did not consistently induce such increases [32]. This decrease in salivary cortisol may represent a calming response during prolonged passive viewing, but may also reflect elapsed time, habituation to the experimental setting, the normal diurnal decline in cortisol, or the task context. The present study cannot distinguish these possible contributions. No interaction was observed between videos with high interest levels (comedy films) and those with low interest levels (lectures), and the significant main effect of time suggests that cortisol levels decreased over time without evidence of a different trajectory between video conditions. However, because viewing duration was fixed at 90 minutes, this finding does not establish that viewing duration influences the HPA axis more than video content. Circadian variation must also be considered, as cortisol generally declines across the waking day [33]. We collected saliva samples between 09:00 and 12:00 or between 13:00 and 17:00, with both viewing sessions conducted at the same time of day for each participant on separate days. This timing was intended to reduce confounding by time of day when comparing video conditions. Nevertheless, matching session times within participants does not eliminate the natural decline in cortisol during each session, and differences in sampling times between participants may contribute to variability in cortisol levels and their changes over time. However, the timing of measures must be considered: HRV reflects rapid autonomic fluctuations, whereas salivary cortisol responds more slowly, with a delay following psychological stress [23]. This temporal lag between immediate cognitive responses and delayed hormonal changes may have contributed to the absence of significant cortisol differences between video conditions despite the clear subjective differences in interest. The sampling schedule may also have limited the detection of transient cortisol responses to specific portions of the videos.

### 3. Participants reported greater fatigue and lower concentration, interest, and comprehension after low-interest than high-interest video viewing

While subjective cognitive responses differed clearly between video conditions, LF differed in average level and HR showed a significant interest × time interaction Participants reported lower concentration and comprehension and greater fatigue during low-interest viewing, whereas no significant interest × time interactions were detected for the other physiological indicators.

To objectify interest in this study, we contrasted comedy and lectures, which maximize subjective engagement. However, these stimuli differ not only in interest but also in emotion, arousal, and cognitive load, which are potential confounding factors. For example, previous research reported only modest associations between physiological responses and subjective emotional experience during emotional film viewing [34]. These findings suggest that subjective engagement and physiological regulation were only partially aligned in this study. This difference in observed patterns does not establish a dissociation between subjective experience and physiological regulation, as limited statistical power, measurement sensitivity, and differences in response timing may also explain the findings. Therefore, the present design does not allow interest to be isolated as a single causal factor. Future research should adopt standardized tools to more clearly separate interest from cognitive load and emotional valence.

### 4. Exploratory analysis of sex differences and real-world implications

The exploratory analysis detected neither a significant main effect of sex nor a significant video condition × sex interaction for the physiological indicators examined. Previous research on sex differences has reported that while men often show increased catecholamine responses, women may exhibit more pronounced heart rate responses to stress [35]. However, a previous study of teachers delivering lectures reported comparable lecture-related reactivity in salivary amylase, CgA, and cortisol between men and women, although sex differences were observed in the cortisol awakening response [36], these differences also may not have been apparent in this study.

Given the small sample and uneven sex distribution, these findings should be interpreted as preliminary and exploratory. The 90-minute viewing protocol in this study was designed to simulate common real-world screen exposure scenarios, such as standard university lectures, feature-length films, or prolonged remote business meetings. Our results suggest that while individuals may experience stress or boredom (subjective/cognitive), their endocrine response, as indexed by salivary cortisol, may decline over time even when subjective fatigue or boredom is reported. The observed difference in patterns of subjective fatigue and physiological responses highlights the complexity of modern digital stress and the importance of using multi-modal indicators (ANS and HPA) to obtain a full picture of human-screen interactions.

### Limitations of the study

Our study had several limitations. First, to maximize the contrast in subjective engagement, two types of videos were used: high-interest (comedy movie) and low-interest (lecture). However, this imposes certain constraints on the definition of interest. Since the same videos were used for all participants, the content of each video did not necessarily align with individual preferences. Furthermore, comedy and lectures differ not only in level of interest but also in emotional value, arousal, and cognitive load. These confounding factors complicate isolating interest as the variable influencing physiological responses. While the contrast chosen here was selected to create a clear experiential difference, future research should quantitatively assess subjective workload using questionnaires such as the NASA Task Load Index (NASA-TLX), alongside separate measures of interest and emotional valence [37]. To clearly distinguish cognitive immersion from physiological stress responses during prolonged video viewing, a wider range of video content and more granular objective metrics are required. Although the 90-minute protocol was intended to represent prolonged viewing in everyday settings, fatigue and reduced attention may have developed during viewing and contributed to the physiological changes. These effects could not be separated from the video content because subjective ratings were obtained only after viewing. These self-reported ratings may also have been influenced by expectations and social desirability. Furthermore, without a non-viewing control condition, such as quiet sitting or an alternative cognitive task, the observed changes cannot be attributed specifically to video viewing or distinguished from changes that might have occurred over the same period without video exposure.

Second, the sample size was small (n = 26), limiting the generalizability of the findings and the statistical power to detect differences. Furthermore, all participants were university students aged 21–24 years, limiting the applicability of the findings to other age groups and populations. Effect estimates and their 95% confidence intervals were reported to describe the magnitude and precision of between-condition differences averaged across time and between-condition differences in post-minus-pre viewing changes (Table 4 and S3 Table). Nevertheless, the small sample may have limited the detection of small effects. Specifically, the uneven sex distribution (10 males, 16 females) further constrains the ability to draw conclusions regarding sex-related differences. Therefore, our findings regarding sex should be considered exploratory. Non-significant interaction effects do not establish equivalent responses between conditions, and uncertainty about small condition-dependent differences remains. No additional power analysis was performed. The confidence intervals describe the precision of the reported contrasts but do not quantify the power to detect interest × time interactions. Future studies should employ larger, more balanced samples to clarify the impact of sex differences during screen exposure. Residual diagnostics showed departures from normality and several large residuals for some outcomes. All verified observations were retained and analyses were performed on the original measurement scale; the robustness of the corresponding model-based estimates, confidence intervals, and significance tests to these departures was not established.

Third, because the saliva samples were collected at three separate time points, we were unable to fully characterize the temporal interrelationship between salivary stress hormones and variations in the autonomic nervous system. Sampling before viewing and at 45 and 90 minutes may have missed rapid or transient endocrine responses. Salivary cortisol and amylase are known to exhibit a time lag following stress [23], making it difficult to analyze their real-time interrelationships with autonomic nervous system variations. Since baseline psychological stress indicators (e.g., STAI, PSS) were not measured, individual differences in the autonomic nervous system and salivary indicators could not be fully corrected. Future research should incorporate psychological measures for each subject and include methods such as simultaneous sampling and analysis. In addition, HPA axis assessment was limited to salivary cortisol; amylase and CgA provided complementary autonomic/SAM-related information rather than direct measures of HPA axis activity. The absence of additional endocrine measures, such as ACTH, limits characterization of HPA axis regulation.

Although participants completed both video conditions on separate days in a randomized order, each condition was assessed only once, preventing evaluation of within-condition test–retest reliability. Randomization was intended to reduce systematic order effects, but does not rule out order or day-to-day influences. Finally, because EEG or neuroimaging was not performed, the peripheral physiological findings do not permit direct conclusions about central nervous system activity.

## Conclusions

This study found that subjective cognitive evaluations (concentration, comprehension, interest, and fatigue) differed between the high-interest and low-interest video conditions during prolonged video viewing. A significant interest × time interaction was detected for HR, whereas no significant interactions were detected for the other physiological indicators. Average levels differed between conditions only for LF.

In both viewing conditions, salivary cortisol decreased over time, although this decrease cannot be separated from the potential effects of diurnal variation and habituation during the sessions. LF/HF, LF, HF, and VLF also changed over time, but none showed a significant interest × time interaction. These findings suggest differing patterns of subjective cognitive experiences and measured physiological responses during prolonged video viewing. As digital environments expand, future research is needed to explore the impact of such cognitive load and physiological indicators on health.

## Supporting information

S1 Table Exploratory analysis of the main effects of video condition and sex and their interaction on physiological indicators. (XLSX)

S2 Table Correlation between subjective scores and physiological indicators. (XLSX)

S3 Table Between-condition differences in changes from pre- to post-viewing in physiological indicators. (XLSX)

S4 Table Raw data of numerical rating scale responses. (XLSX)

S5 Table Raw data of the heart rate variability measures. HR, heart rate; LF, low frequency; HF, high frequency. VLF: very low frequency.
(XLSX)

S6 Table Raw data of the concentrations of salivary hormones. (XLSX)

## References

[1] Kazlauskas E, Quero S. Adjustment and coronavirus: How to prepare for COVID-19 pandemic-related adjustment disorder worldwide? Psychol Trauma. 2020;12(S1):S22–S4. doi: 10.1037/tra0000706 32551753

[2] Van Zoonen W, Sivunen AE. The impact of remote work and mediated communication frequency on isolation and psychological distress. Eur J Work Organ Psychol. 2021;31(4):610–21. doi: 10.1080/1359432x.2021.2002299

[3] Figueiredo S, Joao R, Alho L, Hipolito J. Psychological research on sleep problems and adjustment of working hours during teleworking in the COVID-19 pandemic: An exploratory study. Int J Environ Res Public Health. 2022;19(21):14305. doi: 10.3390/ijerph192114305 36361185 PMC9656353

[4] Neophytou E, Manwell LA, Eikelboom R. Effects of excessive screen time on neurodevelopment, learning, memory, mental health, and neurodegeneration: A scoping review. Int J Ment Health Addict. 2019;19(3):724–44. doi: 10.1007/s11469-019-00182-2

[5] Liu S, Lan Y, Chen B, He G, Jia Y. Smartphone use time and total screen time among students aged 10-19 and the effects on academic stress: A large longitudinal cohort study in Shanghai, China. Front Public Health. 2022;10:869218. doi: 10.3389/fpubh.2022.869218 35655462 PMC9152090

[6] Shaffer F, Ginsberg JP. An overview of heart rate variability metrics and norms. Front Public Health. 2017;5:258. doi: 10.3389/fpubh.2017.00258 29034226 PMC5624990

[7] Billman GE. The LF/HF ratio does not accurately measure cardiac sympatho-vagal balance. Front Physiol. 2013;4:26. doi: 10.3389/fphys.2013.00026 23431279 PMC3576706

[8] Usui H, Nishida Y. The very low-frequency band of heart rate variability represents the slow recovery component after a mental stress task. PLoS One. 2017;12(8):e0182611. doi: 10.1371/journal.pone.0182611 28806776 PMC5555691

[9] Rechlin T, Weis M, Spitzer A, Kaschka WP. Are affective disorders associated with alterations of heart rate variability? J Affect Disord. 1994;32(4):271–5. doi: 10.1016/0165-0327(94)90091-4 7897091

[10] Bair A, Marksteiner J, Falch R, Ettinger U, Reyes Del Paso GA, Duschek S. Features of autonomic cardiovascular control during cognition in major depressive disorder. Psychophysiology. 2021;58(1):e13628. doi: 10.1111/psyp.13628 32621782

[11] Chang JS, Kim EY, Jung D, Jeong SH, Kim Y, Roh M-S, et al. Altered cardiorespiratory coupling in young male adults with excessive online gaming. Biol Psychol. 2015;110:159–66. doi: 10.1016/j.biopsycho.2015.07.016 26253868

[12] Zhong X, Hilton HJ, Gates GJ, Jelic S, Stern Y, Bartels MN, et al. Increased sympathetic and decreased parasympathetic cardiovascular modulation in normal humans with acute sleep deprivation. J Appl Physiol (1985). 2005;98(6):2024–32. doi: 10.1152/japplphysiol.00620.2004 15718408

[13] Binder EB, Nemeroff CB. The CRF system, stress, depression and anxiety-insights from human genetic studies. Mol Psychiatry. 2010;15(6):574–88. doi: 10.1038/mp.2009.141 20010888 PMC3666571

[14] Lac G. Saliva assays in clinical and research biology. Pathol Biol (Paris). 2001;49(8):660–7. doi: 10.1016/s0369-8114(01)00228-0 11692755

[15] Kudielka BM, Buske-Kirschbaum A, Hellhammer DH, Kirschbaum C. HPA axis responses to laboratory psychosocial stress in healthy elderly adults, younger adults, and children: impact of age and gender. Psychoneuroendocrinology. 2004;29(1):83–98. doi: 10.1016/s0306-4530(02)00146-4 14575731

[16] Kirschbaum C, Kudielka BM, Gaab J, Schommer NC, Hellhammer DH. Impact of gender, menstrual cycle phase, and oral contraceptives on the activity of the hypothalamus-pituitary-adrenal axis. Psychosom Med. 1999;61(2):154–62. doi: 10.1097/00006842-199903000-00006 10204967

[17] Nater UM, Rohleder N. Salivary alpha-amylase as a non-invasive biomarker for the sympathetic nervous system: Current state of research. Psychoneuroendocrinology. 2009;34(4):486–96. doi: 10.1016/j.psyneuen.2009.01.014 19249160

[18] Noto Y, Sato T, Kudo M, Kurata K, Hirota K. The relationship between salivary biomarkers and state-trait anxiety inventory score under mental arithmetic stress: A pilot study. Anesth Analg. 2005;101(6):1873–6. doi: 10.1213/01.ANE.0000184196.60838.8D 16301277

[19] Felmingham KL, Tran TP, Fong WC, Bryant RA. Sex differences in emotional memory consolidation: The effect of stress-induced salivary alpha-amylase and cortisol. Biol Psychol. 2012;89(3):539–44. doi: 10.1016/j.biopsycho.2011.12.006 22248928

[20] Kanno T, Asada N, Yanase H, Iwanaga T, Yanaihara N. Salivary secretion of chromogranin A. Control by autonomic nervous system. Adv Exp Med Biol. 2000;482:143–51. doi: 10.1007/0-306-46837-9_11 11192576

[21] Takatsuji K, Sugimoto Y, Ishizaki S, Ozaki Y, Matsuyama E, Yamaguchi Y. The effects of examination stress on salivary cortisol, immunoglobulin A, and chromogranin A in nursing students. Biomed Res. 2008;29(4):221–4. doi: 10.2220/biomedres.29.221 18724010

[22] Fujimoto S, Nomura M, Niki M, Motoba H, Ieishi K, Mori T, et al. Evaluation of stress reactions during upper gastrointestinal endoscopy in elderly patients: Assessment of mental stress using chromogranin A. J Med Invest. 2007;54(1–2):140–5. doi: 10.2152/jmi.54.140 17380025

[23] Obayashi K. Salivary mental stress proteins. Clin Chim Acta. 2013;425:196–201. doi: 10.1016/j.cca.2013.07.028 23939251

[24] Cohen J. Statistical power analysis for the behavioral sciences. New York: Academic Press; 1977. pp. 474.

[25] Nakagawa S, Schielzeth H. A general and simple method for obtaining R 2 from generalized linear mixed‐effects models. Methods Ecol Evol. 2012;4(2):133–42. doi: 10.1111/j.2041-210x.2012.00261.x

[26] Codispoti M, Surcinelli P, Baldaro B. Watching emotional movies: Affective reactions and gender differences. Int J Psychophysiol. 2008;69(2):90–5. doi: 10.1016/j.ijpsycho.2008.03.004 18433903

[27] Wells DL. The effect of videotapes of animals on cardiovascular responses to stress. Stress Health. 2005;21(3):209–13. doi: 10.1002/smi.1057

[28] Hedman A, Hjemdahl P, Nordlander R, Aström H. Effects of mental and physical stress on central haemodynamics and cardiac sympathetic nerve activity during QT interval-sensing rate-responsive and fixed rate ventricular inhibited pacing. Eur Heart J. 1990;11(10):903–15. doi: 10.1093/oxfordjournals.eurheartj.a059612 2265641

[29] Takai N, Yamaguchi M, Aragaki T, Eto K, Uchihashi K, Nishikawa Y. Effect of psychological stress on the salivary cortisol and amylase levels in healthy young adults. Arch Oral Biol. 2004;49(12):963–8. doi: 10.1016/j.archoralbio.2004.06.007 15485637

[30] Sánchez-Navarro JP, Maldonado EF, Martínez-Selva JM, Enguix A, Ortiz C. Salivary alpha-amylase changes promoted by sustained exposure to affective pictures. Psychophysiology. 2012;49(12):1601–9. doi: 10.1111/j.1469-8986.2012.01475.x 23078627

[31] Cook CJ, Crewther BT. Changes in salivary testosterone concentrations and subsequent voluntary squat performance following the presentation of short video clips. Horm Behav. 2012;61(1):17–22. doi: 10.1016/j.yhbeh.2011.09.006 21983238

[32] Budde H, Pietrassyk-Kendziorra S, Bohm S, Voelcker-Rehage C. Hormonal responses to physical and cognitive stress in a school setting. Neurosci Lett. 2010;474(3):131–4. doi: 10.1016/j.neulet.2010.03.015 20226843

[33] Miller R, Stalder T, Jarczok M, Almeida DM, Badrick E, Bartels M, et al. The CIRCORT database: Reference ranges and seasonal changes in diurnal salivary cortisol derived from a meta-dataset comprised of 15 field studies. Psychoneuroendocrinology. 2016;73:16–23. doi: 10.1016/j.psyneuen.2016.07.201 27448524 PMC5108362

[34] Mauss IB, Levenson RW, McCarter L, Wilhelm FH, Gross JJ. The tie that binds? Coherence among emotion experience, behavior, and physiology. Emotion. 2005;5(2):175–90. doi: 10.1037/1528-3542.5.2.175 15982083

[35] Kajantie E, Phillips DIW. The effects of sex and hormonal status on the physiological response to acute psychosocial stress. Psychoneuroendocrinology. 2006;31(2):151–78. doi: 10.1016/j.psyneuen.2005.07.002 16139959

[36] Filaire E, Dreux B, Massart A, Nourrit B, Rama LM, Teixeira A. Salivary alpha-amylase, cortisol and chromogranin A responses to a lecture: impact of sex. Eur J Appl Physiol. 2009;106(1):71–7. doi: 10.1007/s00421-009-0991-z 19190932

[37] Hart SG, Staveland LE. Development of NASA-TLX (Task Load Index): Results of empirical and theoretical research. In: Hancock PA, Meshkati N, editors. Advances in Psychology. North-Holland; 1988. pp. 139–83.

