# Supplementary material for: Cognitive and physiological responses to prolonged video viewing: Activities of the autonomic nervous system and hypothalamo-pituitary-adrenal axis in relation to video interest
Source: PLoS One. 2026 Nov 12;21(11):e0362672. doi: 10.1371/journal.pone.0362672 (PMC13759085; doi:10.1371/journal.pone.0362672)
Supplement: Supplementary file 4 [file pone.0362672.s010.docx]

**Response to Reviewers**

Manuscript ID: PONE-D-25-64004R1

Manuscript title: Cognitive and Physiological Responses to Prolonged Video Viewing: Activities of the Autonomic Nervous System and Hypothalamo-Pituitary-Adrenal Axis in Relation to Video Interest.

(Former title: Impact of prolonged video viewing on physical and mental stress: An analysis of autonomic nervous system activity and salivary stress markers.)

Dear Dr. Yoko Hasegawa

Academic Editor, PLOS ONE

Thank you for your careful evaluation of our manuscript and for your constructive comments. We revised the manuscript to clarify the study design, improve statistical reporting, and interpret the physiological findings more cautiously.

The principal revisions include separate evaluation of LF and HF power, reporting of model-estimated differences and their 95% confidence intervals, clarification of the randomized viewing order and saliva collection times, and expanded discussion of methodological limitations. We have also distinguished average between-condition differences from differences in temporal trajectories and revised statements that could imply causal effects or equivalent responses despite non-significant interactions.

We have carefully considered all points raised by the editor and reviewers. We believe these revisions have significantly improved the manuscript.

Our point-by-point responses are provided below.

Responses to Reviewer #2

Comment 1

In the abstract, the terms ‘high-interest’ and ‘low-interest’ videos are used. However, the content of these videos is not specified. For better clarity, the type of videos should be clearly stated in the abstract. For example, high-interest video (humorous video) should be indicated.

Response:

Thank you for this suggestion. We have specified the video content in the Abstract as high-interest (comedy) and low-interest (lecture) videos. This terminology is consistent with the description of the video conditions in the Methods.

Revised in: Abstract (p. 2, lines 24–25).

Comment 2

The revised abstract is better written than the previous version. The earlier abstract clearly required complete rewriting.

Response:

Thank you for your positive assessment of the revised Abstract. We further refined it to specify the video content, include LF power among the physiological measures, and distinguish observed time effects and average between-condition differences from non-significant interest × time interactions.

Revised in: Abstract (pp. 2–3, lines 24–41).

Comment 3

In this type of study, to minimize the temporal effect, it would be more appropriate to start with low-interest videos in some participants and high-interest videos in others. It is understood that randomizing the video order was intended to eliminate this temporal difference. However, this is not clearly shown in Figure 1.

Response:

We agree that the viewing order required clearer explanation. Each participant completed both video conditions on separate days, and the viewing order was randomized across participants. Some participants viewed the high-interest video first, whereas others viewed the low-interest video first.

We have revised the Methods and Figure 1 to explicitly describe these two sequences. We also clarified that we conducted the sessions at the same time of day for each participant. We intended randomization to reduce systematic order and period effects; however, we now acknowledge that it does not rule out order effects or day-to-day influences.

Revised in: Methods (p. 7, lines 112–114), Fig 1 and Fig 1 legend (p. 7, lines 118–125), Limitations (p. 26, lines 467–470).

Comment 4

In the discussion, it is stated that the LF/HF parameter is controversial as a marker of sympathetic activity. This is correct; however, when only the LF/HF ratio is considered, it is important to determine whether the change is due to LF or HF components. Therefore, LF and HF parameters should also be evaluated separately. After mentioning that it is controversial, it would be more appropriate to interpret your findings based on the changes in LF and HF and explain whether they reflect a relaxed state.

Response:

Thank you for this important comment. We have added the LF analysis and now report LF and HF separately, together with LF/HF.

Both LF and HF showed significant main effects of time and increased during viewing. Thus, LF/HF decreased alongside increases in both components rather than a decrease in LF. We have revised the Discussion to explain that this pattern may reflect relative changes in the two components.

Across measurement time points, LF was higher in the high-interest condition, whereas HF showed no significant main effect of interest. The higher LF/HF ratio in the high-interest condition was therefore consistent with the observed LF difference, although the relative contributions of LF and HF to the ratio were not directly quantified. Furthermore, no significant interaction was observed between the variables of interest and time for either LF or HF.

We also noted that LF/HF is not a specific measure of sympathetic activity and that HF is influenced by respiration and prevailing heart rate. The decrease in heart rate and increase in HF may be consistent with increased cardiac vagal modulation and a more relaxed physiological state, but they do not establish parasympathetic activation or relaxation.

Revised in: Abstract (p. 2, lines 27–34), Introduction (pp. 4-5, lines 54–65), Methods (p. 8, lines 130–138), Results (p. 16, lines 257–268), Table 2 (p. 13), Table 3 (p. 14), Table 4 (p. 15), Fig 3, Discussion (pp. 19–20, lines 329–345) and Supporting Information (S1-3 Table).

Comment 5

In the discussion, the decrease in cortisol levels is explained only in terms of video duration. This suggests a methodological weakness, because the aim of the study was not to investigate the effect of video duration.

Response:

We agree and have revised the interpretation. Because viewing duration was fixed at 90 minutes, the present study cannot determine the effect of varying video duration or establish that duration has a greater influence than video content.

The revised Discussion considers several possible contributors to the cortisol decrease, including elapsed time, habituation to the experimental setting, normal diurnal decline, and task context. We explicitly state that the present design cannot distinguish these contributions and that the decrease does not, by itself, establish reduced physiological stress attributable to video viewing.

Revised in: Discussion (p. 21, lines 357–376) and Conclusions (p. 26, lines 478–480).

Responses to Reviewer #3

Comment 1

The sample size is insufficient: The study includes only 26 participants, which limits statistical power and reduces the generalizability of the findings.

Response:

We agree that the sample size limits both statistical power and generalizability. We have explicitly acknowledged these limitations and the possibility that small effects were not detected.

To improve interpretation of the findings, we report model-estimated between-condition differences and their 95% confidence intervals in Table 4 and post-minus-pre viewing changes and their 95% confidence intervals in S3 Table. These estimates describe the magnitude and precision of the reported contrasts, but they do not resolve the limitations of the small sample. We also state that non-significant interactions do not establish equivalent responses between conditions.

Revised in: Methods (p. 10, lines 172–181), Results (p. 12, lines 222–224), Table 4 and its legend (pp. 15–16), Limitations (p. 25, lines 442–454), and Supporting Information (S3 Table).

Comment 2

Limited population diversity: All participants are university students within a narrow age range (21–24 years), limiting the applicability of the findings to broader and more diverse populations.

Response:

As you pointed out, the age range for this initiative is limited. The Limitations now explicitly state that all participants were university students aged 21–24 years and that the findings may not be applicable to other age groups or populations. Accordingly, the conclusions are restricted to the responses observed in the present sample and viewing conditions.

Revised In: Limitations (p. 25, lines 442–444).

Comment 3

The experimental duration is excessively long: A 90-minute viewing period may induce mental fatigue and reduced attention, confounding the effects of the independent variable.

Response:

We selected the 90-minute protocol to reflect prolonged viewing in everyday settings, such as university lectures and feature-length films. Nevertheless, we agree that fatigue and reduced attention may have developed during viewing and contributed to the physiological changes.

We added this limitation and clarified that these contributions cannot be separated from those of video content because we obtained subjective ratings only after viewing. We have also revised the description of the subjective findings to refer to differences between conditions after viewing, rather than implying changes from pre-viewing levels.

Revised in: Discussion (p. 22, lines 383–390) and Limitations (p. 24, lines 434–438).

Comment 4

Time effects are not adequately controlled: It is unclear whether the observed physiological changes are due to video content or simply prolonged exposure over time.

Response:

We agree that a significant main effect of time cannot identify the cause of the physiological changes. We have revised the Discussion to acknowledge that elapsed time, habituation, prolonged seated rest, and, for cortisol, diurnal decline may have contributed.

We also distinguish average between-condition differences from differences in temporal trajectories. No significant interest × time interactions were detected. Furthermore, because the average condition estimates include pre-viewing measurements, we clarify that they do not necessarily represent differences induced by video viewing.

Revised in: Discussion (pp. 18–19, lines 305–312; p. 21, lines 362–376), Limitations (pp. 24–25, lines 438–441), and Conclusions (p. 26, lines 478–480).

Comment 5

Lack of a proper control condition: The absence of control conditions (e.g., passive rest, quiet sitting, or alternative cognitive tasks) weakens causal interpretation.

Response:

Thank you for this important comment. We have explicitly acknowledged the absence of a non-viewing control condition, such as quiet sitting or an alternative cognitive task.

The revised manuscript states that the observed changes cannot be attributed specifically to video viewing or distinguished from changes that might have occurred over the same period without video exposure. We have also revised the interpretation and conclusions to avoid attributing the physiological findings solely to the videos.

Revised in: Introduction (p. 5, lines 83–85), Discussion (p. 19, lines 310–312; p. 21, lines 357–365), Limitations (pp. 24–25, lines 438–441), and Conclusions (p. 26, lines 478–480).

Comment 6

Limited sensitivity of physiological measures: Similar ANS and HPA responses in both high- and low-interest conditions raise concerns about the sensitivity of the measurements.

Response:

We agree that non-significant differences may reflect limited measurement sensitivity, sampling timing, or statistical power. We now discuss these alternative explanations and avoid interpreting the findings as evidence that the physiological responses were equivalent.

The results also warrant distinction between average levels and temporal trajectories: HR, LF/HF, LF, and VLF showed significant average between-condition differences, whereas no physiological indicator showed a significant interest × time interaction. Table 4 provides the corresponding estimates and confidence intervals. For non-significant contrasts, the confidence intervals illustrate the remaining uncertainty rather than establishing the absence of a physiological response.

Revised in: Results (p. 16, lines 252–268; p. 17, lines 269–277), Table 4 and its legend (pp. 15–16), Discussion (p. 20, lines 346–353; pp. 22–23, lines 396–399), and Limitations (p. 25, lines 449–453).

Comment 7

Oversimplified interpretation of HRV parameters: The study interprets LF/HF ratio and HF power as indicators of autonomic balance without acknowledging ongoing debates about their validity.

Response:

Thank you for highlighting this issue. We have revised the Introduction, Methods, and Discussion to acknowledge the limitations of HRV interpretation.

LF/HF is now described as a frequency-domain HRV measure rather than a direct measure of sympathetic activity or sympathovagal balance. We clarify that LF reflects multiple regulatory influences and that HF is associated with cardiac vagal modulation but is also influenced by respiration and prevailing heart rate. We interpret LF and HF separately and in conjunction with heart rate, and we avoid concluding that the observed pattern establishes sympathetic withdrawal, parasympathetic activation, or relaxation.

Revised in: Introduction (pp. 4-5, lines 54–65), Methods (p. 8, lines 133–138), and Discussion (pp. 19–20, lines 329–345).

Comment 8

Insufficient endocrine assessment: Only cortisol, amylase, and chromogranin A were measured, which provides a limited evaluation of the Hypothalamic-Pituitary-Adrenal Axis.

Response:

We agree with that point and have clarified the roles of these markers. HPA axis assessment in this study was limited to salivary cortisol. Amylase and CgA provided complementary autonomic/SAM-related information rather than direct measures of HPA axis activity.

We acknowledge that the absence of additional endocrine measures, such as ACTH, limits characterization of HPA axis regulation.

Revised in: Limitations (p. 26, lines 463–466).

Comment 9

Circadian rhythm of cortisol not addressed: Cortisol levels naturally fluctuate throughout the day, yet timing and control for circadian variation are not clearly discussed.

Response:

We have clarified the sampling schedule and its limitations. Saliva samples were collected between 09:00 and 12:00 or between 13:00 and 17:00. For each participant, the two sessions were conducted at the same time of day on separate days, with corresponding samples collected at matched times.

This procedure was intended to reduce time-of-day confounding in comparisons between conditions. However, we explicitly acknowledge that matching session times does not eliminate natural cortisol decline within each session and that differences in sampling times between participants may contribute to variability. We have added a reference supporting diurnal cortisol decline and included this alternative explanation in the Discussion and Conclusions.

Revised in: Methods (p. 8, lines 141–146), Discussion (p. 21, lines 369–376), and Conclusions (p. 26, lines 478–480).

Comment 10

Weak interpretation of subjective–objective dissociation: The conclusion that subjective experience and physiological responses are dissociated is not sufficiently supported, as alternative explanations are not ruled out.

Response:

We agree with this perspective and have moderated this interpretation. The revised manuscript describes differing patterns of subjective ratings and measured physiological responses rather than an established dissociation.

We explicitly acknowledge that limited statistical power, measurement sensitivity, and differences in response timing may explain the findings. We also recognize that the comedy and lecture videos differed in emotional valence, arousal, and cognitive load, preventing interest from being isolated as a single causal factor.

Revised in: Abstract (p. 3, lines 39–41), Discussion (pp. 22–23, lines 386–402) and Conclusions (p. 26, lines 474–482).

Comment 11

Potential bias in subjective measures: Self-reported ratings are vulnerable to social desirability and expectation bias.

Response:

Thank you for your comment. We have added a statement acknowledging that the subjective ratings may have been influenced by participants’ expectations and social desirability. These ratings are interpreted as self-reported evaluations rather than objective measures of cognitive performance.

Revised in: Limitations (p. 24, lines 437–438).

Comment 12

Limited statistical reporting: Effect sizes and power analysis are not reported, limiting the interpretation of the findings.

Response:

We have expanded the statistical reporting. Table 1 reports effect sizes for the paired comparisons of subjective ratings. Table 3 reports marginal and conditional R² for each mixed model, and the Methods and Discussion clarify that these represent model-level explained variance rather than the magnitude of individual fixed effects.

Table 4 reports model-estimated between-condition differences averaged across measurement time points and their 95% confidence intervals. S3 Table reports post-minus-pre viewing changes averaged across video conditions and their 95% confidence intervals.

No additional power analysis was performed. We explicitly acknowledge this and clarify that the confidence intervals describe the precision of the reported contrasts but do not quantify power to detect interest × time interactions. Therefore, sample-size limitations remain part of the interpretation.

Revised in: Methods (pp. 9–10, lines 168–181), Results (p. 12, lines 222–224), Table 1 (p. 12), Table 3 and its legend (pp. 14–15), Table 4 and its legend (pp. 15–16), Discussion (p. 19, lines 313–316), Limitations (p. 25, lines 444–453), and Supporting Information (S3 Table).

Comment 13

Interpretation despite non-significant interaction effects: Conclusions about content-related differences are made despite the absence of significant interaction effects.

Response:

We agree that condition main effects and condition × time interactions must be interpreted separately. We have revised the Abstract, Results, Discussion, and Conclusions accordingly.

The manuscript now distinguishes the average between-condition differences observed for HR, LF/HF, LF, and VLF from the absence of significant differences in temporal trajectories. We do not interpret the non-significant interactions as evidence of equivalent responses. We also acknowledge that the average condition estimates include pre-viewing measurements and do not necessarily represent differences induced by video viewing.

Revised in: Abstract (pp. 2–3, lines 33–41), Results (p. 16, lines 254–268; p. 17, lines 269–277), Table 4 legend (p. 16, lines 247–250), Discussion (pp. 18–19, lines 305–310), Limitations (p. 25, lines 449–453), and Conclusions (p. 26, lines 474–482).

Comment 14

Timing of biomarker collection may be suboptimal: Salivary sampling points may not adequately capture rapid or transient physiological changes.

Response:

We appreciate this important point. We clarified that we collected saliva immediately before viewing, 45 minutes after viewing began, and immediately after completing the 90-minute video.

The revised Discussion and Limitations acknowledge that this schedule may have missed rapid or transient responses to particular portions of the videos and cannot fully characterize the temporal relationship between salivary markers and HRV. We also discuss the delayed cortisol response relative to rapid autonomic fluctuations.

Revised in: Methods (p. 8, lines 141–143), Discussion (pp. 21–22, lines 376–381), and Limitations (p. 25, lines 455–460).

Comment 15

Single-session study design: The use of a single session increases inter-individual variability and reduces reliability.

Response:

We apologize that the original description did not sufficiently clarify the design. Each participant completed two sessions on separate days, one for each video condition, in randomized order. The main repeated-measures analyses included subject-specific random intercepts to account for within-participant correlation.

Nevertheless, we assessed each condition only once. We therefore agree that within-condition test–retest reliability could not be evaluated. We clarified the two-session design and added this limitation, including potential order and day-to-day influences.

Revised in: Methods (p. 7, lines 110–114), Fig 1 and Fig 1 legend (p. 7, lines 118–125), and Limitations (p. 26, lines 467–470).

Comment 16

Lack of central nervous system measures: The absence of neurophysiological methods (e.g., EEG or neuroimaging) limits interpretation at the brain level.

Response:

We agree with that point. We have added a statement acknowledging that EEG and neuroimaging were not performed and that the peripheral physiological findings do not permit direct conclusions about central nervous system activity.

Revised In: Limitations (p. 26, lines 470–471).

We hope these revisions have adequately addressed the Editor’s and Reviewers’ concerns, and we are grateful for the thoughtful comments, which helped us improve the manuscript's clarity and rigor.

Sincerely,

Hiroki Matsui, Ph.D.

Department of Laboratory Sciences,

Gunma University Graduate School of Health Sciences
