# Supplementary material for: Cognitive and physiological responses to prolonged video viewing: Activities of the autonomic nervous system and hypothalamo-pituitary-adrenal axis in relation to video interest
Source: PLoS One. 2026 Nov 12;21(11):e0362672. doi: 10.1371/journal.pone.0362672 (PMC13759085; doi:10.1371/journal.pone.0362672)
Supplement: Supplementary file 1 [file pone.0362672.s007.docx]

**Response to Reviewers**

Manuscript title: Impact of prolonged video viewing on physical and mental stress: An analysis of autonomic nervous system activity and salivary stress markers

Manuscript ID: PONE-D-25-04885

Corresponding author: Hiroki Matsui

We sincerely thank the Editor and both reviewers for their thorough evaluation and constructive suggestions. We carefully revised the manuscript in accordance with all comments. Below, we provide a point-by-point response. Reviewer comments are shown in italics, followed by our responses.

**Responses to Reviewer #1**

Abstract

1. *“Should mention what were the stress markers measured in the study.”*

Response: We added explicit descriptions of the three salivary markers—cortisol, amylase, and chromogranin A—in the Abstract. (p2, lane 30)

2. *“Explain more clearly about low/high sym/parasym activity.”*

Response: We revised the Abstract to clarify that HF represents parasympathetic activity and LF/HF reflects sympathetic activity. (p2, lanes 26-29)

3. *“Results/conclusion unclear if several results are not significant.”*

Response: We rephrased the results and conclusions to avoid implying significance where none exists and added clarifying statements noting that many physiological differences were not statistically significant. (p2, lanes 35-42)

Introduction

4. *“Should indicate what are the devices.”*

Response: We added specific descriptions of electronic devices used for prolonged viewing. (p4, lanes 47-48)

5. *“Explain how depressive symptoms are associated with autonomic activity.”*

Response: To clarify the relationship between autonomic nervous system indicators and psychiatric symptoms, the following description regarding changes in LF and HF and their association with psychiatric symptoms has been added. (p4, lanes 60-63)

“Increases in LF and sympathetic dominance over heart rate are associated with panic disorder, whereas decreased HF indices and reduced parasympathetic activity are observed in depressive disorders, indicating that autonomic activity indices are correlated with psychiatric symptoms.”

6. *“What kind of visual information?”*

Response: We verified the citation of the referenced paper and added a note specifying that the visual information is from a video game. (p4, lanes 64)

7. *“Elaborate more in the last paragraph about the current study.”*

Response: As you pointed out, in the final paragraph of the abstract, we expanded the rationale for the research and clarified its background and objectives. (p5-6, lanes 80-89)

8. *“It seems related to TV watching more than digital devices.”*

Response: In the final paragraph of the Introduction, we noted that existing research has identified stress associated with ergonomic factors, such as close viewing distances and postural strain when using digital devices like smartphones and tablets. We also clearly stated that this study used a different, standardized offline viewing method with monitors and investigated the impact of two video content types and their varying levels of interest on stress. (p5-6, lanes 80-89)

Methods

9. *“Table 1 not cited; information duplicated.”*

Response: The content of Table 1 was referenced in the main text (p6, lanes 93-94) and has been removed. The current Table 1 differs from the content of the previous Table 1.

10. *“Multiple paired t-test concern due to repeated measures.”*

Response: We revised the statistical analysis method and evaluated each metric based on the two video viewings. For each subject, the area under the curve was calculated using trapezoidal integration, and the AUC values obtained under the two conditions were compared using a paired t-test. The updated analysis method is described in the statistical analysis section. (p8-9, lanes 149-152).

Results & Discussion

11. *“Fig 2 and Table 2 contain similar information.”*

Response: First, Table 2 in the original manuscript corresponds to Table 1 in the revised manuscript. In the revised manuscript, based on comments from other reviewers, we calculated and included the Wilcoxon test statistic Z and the effect size r. We also described the results in the Results section based on the effect size findings (p10, lanes 173-174).

12. *“Be careful reporting non-significant results.”*

Response: In the Abstract, Results, and Discussion sections, descriptions of statistically non-significant parts were revised to avoid expressions implying significance (e.g., p2, lanes 35-36, p3 lanes 39-41). Furthermore, measures that did not show statistically significant differences were rephrased to describe tendencies rather than effects (e.g., p. 12, lanes 201-208; p. 14, lanes 223-230).

13. *“Sex differences: sample too small to conclude.”*

Response: Regarding sex differences, we conducted a two-way ANOVA to examine interaction effects across indicators and tested simple main effects for gender. No significant differences were found. Therefore, we revised the text to describe only tendencies (p12, lanes 204-212; p14, lanes 231-238). In the discussion section as well, we avoided definitive statements about gender differences. Instead, we focused solely on describing the trends and possibilities observed in these results and the prospects for future research (p18-19, lanes 300-322).

**Responses to Reviewer #2**

Methodology

1. *“Sample size small; no power analysis.”*

Response: We added a post-hoc power analysis (G*Power) to the Limitations section and noted that future studies with larger samples are needed (p19-20, lanes 331-338).

2. *“Confounding factors (stress level, caffeine, sleep, video exposure) not controlled.”*

Response: Participants were instructed to avoid caffeine and alcohol before the test, ensure sufficient sleep, and refrain from watching videos immediately before the test to prevent interference with autonomic nervous system activity, as specified in the methods. It was also noted that participants were instructed to avoid eating, drinking, brushing their teeth, and strenuous exercise before measurement to prevent effects on salivary hormone levels. (p6, lanes 96-99) Furthermore, we acknowledged the presence of residual confounding factors and included this as a limitation (p19, lanes 325-330; p20, lanes 343-346).

3. *“High/low interest video selection subjective.”*

Response: We have added details to the Methods section explaining how the two videos were selected and verified by independent students. (p6-7, lanes105-110) We clarified the limitations arising from subjective differences in interest and documented these in the “Limitations” section. (p19, lanes 325-330)

Statistical analysis

4. *“Normality test unclear; effect sizes not reported.”*

Response: As instructed, we performed the Shapiro-Wilk test to assess normality. Since the results showed some data met normality assumptions while others did not, we conducted a nonparametric Wilcoxon signed-rank test on the paired data. The effect size (r) for the Wilcoxon test was calculated using Cohen's reports. These details are described in the Methods section. (p8, lanes 145-148) Additionally, the test's effect size was added to Table 1, and a description of the effect size was also included in the results. (p10, lanes 173-174).

5. *“HRV analysis limited (no RMSSD, total power, VLF).”*

Response: We added VLF analysis and clarified its exploratory nature. (p4, lanes 59-63, Figure 3, Table 2, p17, lanes 271-274). Regarding total power and RMSSD, these parameters are not included in the device's measurement and analysis items (MemCalc/Bonaly Light, GMS, Japan). We explored alternative parameters and analysis methods, but unfortunately, we were unable to perform the analysis. We kindly ask for your understanding that we were unable to include these results in this study.

6. *“Sex differences overstated; no interaction effects tested.”*

Response: Regarding sex differences, we conducted a two-way ANOVA to examine interaction effects across indicators and tested simple main effects for gender. No significant differences were found. Therefore, we revised the text to describe only tendencies (p12, lanes 204-212; p14, lanes 231-238). In the discussion section as well, we avoided definitive statements about gender differences. Instead, we focused solely on describing the trends and possibilities observed in these results and the prospects for future research (p18-19, lanes 300-322).

Conceptual framework and interpretation

7. *“Insufficient engagement with literature on screen time, cognitive load, stress biomarkers. Lower stress but increased autonomic reactivity.”*

Response: Thank you for your critical comments. Based on the relevant literature, we have clarified the distinctions among cognitive engagement, visual stimuli, stress physiology, and prolonged screen exposure, and revised the discussion section accordingly. (p16-18, line 258-298)

Figures & tables

8. *“Figures need error bars.”*

Response: Following PLOS ONE guidelines, the median and interquartile range (IQR) are uniformly displayed as error bars in all figures.

9. *“Tables inconsistent in data format.”*

Response: To maintain consistency across all tables, we have revised the notation to use the median and interquartile range.

10. *“p-values need standardization.”*

Response: We revised the notation for p-values to ensure consistent presentation across all figures and tables.

**Editorial Requirements**

1. “*Manuscript meets PLOS ONE's style requirements”*

Response: We have verified the style requirements of PLOS ONE and checked and revised the entire manuscript to ensure compliance.

2. “*Funder Role Statement”*

Response: We have added the following text to the Sources of Funding section.

“The funders had no role in study design, data collection and analysis, decision to publish, or preparation of the manuscript. “

3. “*Data Availability”*

Response: We confirmed that all raw data required to reproduce results have been uploaded as Supporting Information (S2–S4 Tables).

4. “Citation of Table 1”

Response: The content of Table 1 was referenced in the main text and has been removed. The current Table 1 differs from the content of the previous Table 1.

**Closing statement**

We appreciate the insightful comments that greatly improved the clarity and rigor of our manuscript. All revisions have been incorporated into the revised manuscript and the tracked-changes version. We hope the revised submission meets the standards for publication in PLOS ONE.

Sincerely,

Hiroki Matsui, PhD

Department of Laboratory Sciences

Gunma University Graduate School of Health Sciences
