# Supplementary material for: Cognitive and physiological responses to prolonged video viewing: Activities of the autonomic nervous system and hypothalamo-pituitary-adrenal axis in relation to video interest
Source: PLoS One. 2026 Nov 12;21(11):e0362672. doi: 10.1371/journal.pone.0362672 (PMC13759085; doi:10.1371/journal.pone.0362672)
Supplement: Supplementary file 2 [file pone.0362672.s008.docx]

**Response to Reviewers**

Manuscript ID: PONE-D-25-64004

Manuscript title: Cognitive and Physiological Responses to Prolonged Video Viewing: Activities of the Autonomic Nervous System and Hypothalamo-Pituitary-Adrenal Axis in Relation to Video Interest.

(Former title: Impact of prolonged video viewing on physical and mental stress: An analysis of autonomic nervous system activity and salivary stress markers.)

Dear Dr. Yoko Hasegawa

Academic Editor, PLOS ONE

We sincerely appreciate your thoughtful and constructive comments on the manuscript titled “Cognitive and Physiological Responses to Prolonged Video Viewing: Autonomic Nervous System and Hypothalamo-Pituitary-Adrenal Axis Activity Related to Interest in Videos” (formerly titled: Effects of Prolonged Video Viewing on Physical and Mental Stress: Analysis of Autonomic Nervous Activity and Salivary Stress Markers).

We have carefully considered all points raised by the editor and reviewers. We believe these revisions have significantly improved the manuscript. Our responses to each comment are provided below.

Responses to Academic Editor

Comment 1. Definition of “Stress”

The data (lower HR, higher HF, lower cortisol) point more toward relaxation than stress. Clarify the conceptual focus.

Response:

We appreciate this important point. We agree that the original wording overly emphasized “stress” and did not sufficiently distinguish between physiological stress, cognitive engagement, and subjective experience. Therefore, we clarified that while this study evaluates autonomic nervous system (ANS) and hypothalamo-pituitary-adrenal (HPA) axis-related physiological responses during prolonged video viewing, subjective assessments reflect cognitive and experiential aspects such as concentration, comprehension, interest, and fatigue. We also revised the Discussion section to interpret the decreases in heart rate and the LF/HF ratio, together with the increase in HF and the reduction in salivary cortisol, as findings consistent with relative relaxation rather than increased physiological stress. Furthermore, we added an explanation in the introduction regarding the distinct roles and temporal dynamics of the HPA axis and the sympathetic-adrenal medullary (SAM) system.

Revised In: Title (p 1, lines 1-3), Abstract (p 2-3, lines 19–41), Introduction (p 4, lines 50–52; p5-6, lines 64-86), Discussion (p 14-18, lines 263–317), and Conclusions (p 21-22, lines 387–392).

Comment 2. Definition of “Interest”

Using comedy vs. lectures involves confounding factors (emotion, arousal). Explain the choice and discuss limitations.

Response:

As you pointed out, we have clarified the reasons for selecting comedy videos and lecture videos. The rationale for using contrast videos was to maximize differences in subjective engagement under a controlled, extended viewing protocol. Simultaneously, we explicitly stated that these stimuli differ not only in interest but also in emotional valence, arousal, and cognitive load. Thus, we did not isolate “interest” as the sole causal factor. We added these points to both the Discussion and Limitation sections and propose incorporating standardized measurement methods, such as cognitive load and immersion scales, into future analyses.

Revised In: Discussion (p 19, lines 325–333), and Limitations (p 20, lines 353–363).

Comment 3. Statistical Analysis

Use mixed-effects models to handle within-subject variability.

Response:

Thank you for your suggestion. In response, we reanalyzed the repeated-measures physiological outcomes using a linear mixed-effects model. We included interest in the video and time as fixed effects, along with their interaction, and accounted for within-subjects correlation by including a subject-specific random intercept. We also added post-hoc comparisons based on the fitted model, reporting estimated mean differences and 95% confidence intervals. To assess model fit, we included marginal R² and conditional R² values. The results are presented in Table 3 and the Results section, with interpretations discussed in the Discussion.

Revised In: Methods (p 9, lines 154–160), Results (p 11, lines 199–201; p 14-15, lines 222–249), Discussion (p 16-18, lines 271–317), and Table 3.

Comment 4. Timing of Measures

Discuss the timing mismatch between HRV and salivary cortisol.

Response:

We agree with this perspective and have added clarification to the Discussion section (Section 2). While HRV reflects relatively rapid fluctuations in the autonomic nervous system, salivary cortisol changes more gradually and may reflect physiological responses to psychological events that occurred earlier. This temporal lag contributes to the discrepancy between subjective scores and endocrine markers.

Revised In: Discussion (p 18, lines 311–317) and Limitations (p 21, lines 374–381).

Comment 5. Sample Size and Sex Balance

State that sex analyses are exploratory due to small sample size.

Response:

This paper explicitly states that, due to the small sample size and uneven sex distribution, sex-related analyses should be interpreted cautiously as exploratory. Furthermore, in the “Discussion” and “Limitations” sections, we explicitly stated that our analysis of sex differences is preliminary and exploratory. As shown in Fig. 2–4, we removed the sex-specific figures and presented only the results for the overall sample.

Revised In: Results (p 15, lines 250–251), Discussion (p 19, lines 336–341), Limitations (p 20-21, lines 364–373), and Fig. 2-4.

Comment 6. Additional Considerations (LF/HF, VLF, RMSSD)

Response:

We have addressed the points you raised as follows. First, we supplemented the Discussion section to note that while LF/HF is widely used, its status as a pure indicator of sympathetic activity remains debatable. Second, we added a concise explanation regarding VLF and its physiological significance during prolonged viewing. Third, we added interpretation linking the 90-minute viewing protocol to real-life scenarios such as university lectures and remote meetings. Fourth, we added a correlation analysis between subjective and physiological indicators, summarizing the results in S2 Table.

Regarding RMSSD, since this study primarily used device outputs centered on frequency-domain HRV measurements, the revised manuscript maintains the frequency-domain framework. To clearly indicate this to readers, we added the following sentence to the method section: Time-domain HRV parameters, including RMSSD, were not analyzed because they were not provided by the measurement device used in this study.

Revised In: Methods (p 7-8, lines 127–129), Results (p 15, lines 253–261), Discussion (p 17, lines 289–295; p 19, lines 342-350), Limitations (p 19-20, lines 342–350), and Supporting Information (Table S2).

**Responses to Reviewer #1**

Comment 1. Title: Avoid “physical and mental stress” and incorporate ANS/HPA and video interest.

Response:

We appreciate this valuable suggestion. We agree that the previous title overemphasized what could be inferred from the measured physiological indicators. Therefore, we have revised the title to focus on the ANS and HPA axis activity and to reflect the role of interest in the video. We have also removed the phrase “An analysis of,” as you suggested.

Revised In**:** Title (p 1, lines 1–3).

Comment 2. The purpose, results, and conclusion should better reflect the high-interest versus low-interest design.

Response:

We revised the abstract, introduction, discussion, and conclusion to ensure the distinction between high-interest and low-interest videos is more clearly described throughout the manuscript. Simultaneously, we clarified that although subjective reactions varied with video interest level, no significant interaction between interest level and time was observed for physiological reactions.

Revised In: Title (p 1, lines 1-3), Abstract (p 2-3, lines 19–41), Introduction (p 4, lines 50–52; p5-6, lines 64-86), Discussion (p 14-18, lines 263–317; p 19, lines 325–333), and Conclusions (p 21-22, lines 387–392).

3. Comment 3. The conclusion was too broad and should more directly reflect ANS and HPA findings

Response:

As you pointed out, we revised the conclusion section to directly reflect the data. The revised conclusion states that while interest in the video influenced subjective cognitive evaluations, physiological responses were generally similar across conditions. Salivary cortisol decreased over time in both conditions, indicating reduced HPA axis activity. Meanwhile, HRV indicators changed over time, but no clear dependence on interest levels was observed.

Revised In**:** Conclusions (p 21-22, lines 384–392).

Comment 4. Abstract typo: cortisol was written twice, and the last term should be CgA

Response:

Thank you for pointing out this error. We corrected the Abstract accordingly.

Revised In**:** Abstract (p 2, lines 35–36).

Comment 5. Please make the conclusion firmer regarding which stress axis changed and in what way

Response:

As you pointed out, to more clearly indicate the direction of the physiological findings, we revised both the opening section of the discussion and the conclusion section. Specifically, we explicitly stated that while no clear interest-dependent changes were observed in autonomic nervous system/adrenal cortex system (ANS/SAM) markers, the HPA axis marker, salivary cortisol, decreased over time under both viewing conditions.

Revised In**:** Discussion (p. 16, lines 264–267) and Conclusions (p 21-22, lines 384–392).

Comment 6. The tables are confusing, and a sex-separated presentation is unnecessary

Response:

We agree with this practical suggestion. We simplified the main tables and focused the study's main presentation on intra-subject comparisons and mixed-model results (Tables 1-3). Unnecessary sex-stratified presentations were removed. Instead, sex-related analyses were concisely summarized in the main text, with detailed results retained in supplementary information (Table S1). Additionally, sex-specific results in Figures 2–4 have been removed, and only the overall sample results are presented.

Revised In**:** Tables 1–3, Results (p 11-15, lines 192–261), Fig. 2-4, and Supporting Information (Table S1).

Comment 7. The Discussion should begin by stating what was done and what was found

Response:
As you pointed out, we revised the opening paragraph of the discussion section. We restructured it to first concisely summarize the study design and key findings, then transition to the interpretation by theme. We also reorganized the discussion's structure using clearer subheadings.

Revised In**:** Discussion (p. 16-19, lines 264–333).

Comment 8: Time-domain HRV parameters were not included.

Response:

Thank you for your comment. Since this study was conducted using device outputs centered on frequency-domain HRV indices, we adopted LF/HF, HF, and VLF as the primary HRV indices. Furthermore, in the Discussion section, we clarified that these indices are sufficient to support physiological interpretations. Regarding RMSSD, to indicate that it was not output by the device used in this study, we added the following sentence to the Methods section:

Time-domain HRV parameters, including RMSSD, were not analyzed because they were not provided by the measurement device used in this study.

Revised In**:** Methods (p 7-8, lines 127–129), and Discussion (p. 16-17, lines 269–301).

We hope that these revisions have adequately addressed the Editor’s and Reviewer’s concerns, and we are grateful for the thoughtful comments, which have helped us improve the clarity and rigor of the manuscript.

Sincerely,

Hiroki Matsui, Ph.D.

Department of Laboratory Sciences,

Gunma University Graduate School of Health Sciences
