# Supplementary material for: Cognitive and physiological responses to prolonged video viewing: Activities of the autonomic nervous system and hypothalamo-pituitary-adrenal axis in relation to video interest
Source: PLoS One. 2026 Nov 12;21(11):e0362672. doi: 10.1371/journal.pone.0362672 (PMC13759085; doi:10.1371/journal.pone.0362672)
Supplement: Supplementary file 3 [file pone.0362672.s009.docx]

The study investigated the effects of high-interest (humorous video) and low-interest (educational video) content on stress parameters. The topic of the study is interesting. The manuscript has improved after the necessary revisions and appears stronger. However, there are still methodological concerns in the study.

1- In the abstract, the terms ‘high-interest’ and ‘low-interest’ videos are used. However, the content of these videos is not specified. For better clarity, the type of videos should be clearly stated in the abstract. For example, high-interest video (humorous video) should be indicated.

2- The revised abstract is better written than the previous version. The earlier abstract clearly required complete rewriting.

3- In this type of study, to minimize the temporal effect, it would be more appropriate to start with low-interest videos in some participants and high-interest videos in others. It is understood that randomizing the video order was intended to eliminate this temporal difference. However, this is not clearly shown in Figure 1.

“4- In the discussion, it is stated that the LF/HF parameter is controversial as a marker of sympathetic activity. This is correct; however, when only the LF/HF ratio is considered, it is important to determine whether the change is due to LF or HF components. Therefore, LF and HF parameters should also be evaluated separately. After mentioning that it is controversial, it would be more appropriate to interpret your findings based on the changes in LF and HF and explain whether they reflect a relaxed state.

5- In the discussion, the decrease in cortisol levels is explained only in terms of video duration. This suggests a methodological weakness, because the aim of the study was not to investigate the effect of video duration.
