# Supplementary material for: Cognitive and physiological responses to prolonged video viewing: Activities of the autonomic nervous system and hypothalamo-pituitary-adrenal axis in relation to video interest
Source: PLoS One. 2026 Nov 12;21(11):e0362672. doi: 10.1371/journal.pone.0362672 (PMC13759085; doi:10.1371/journal.pone.0362672)
Supplement: Supplementary file 5 [file pone.0362672.s011.docx]

**Response to Reviewers**

Manuscript ID: PONE-D-25-64004R2

Manuscript title: Cognitive and Physiological Responses to Prolonged Video Viewing: Activities of the Autonomic Nervous System and Hypothalamo-Pituitary-Adrenal Axis in Relation to Video Interest.

Dear Dr. Yoko Hasegawa

Academic Editor, PLOS ONE

Thank you for your careful evaluation of our revised manuscript. In response to your request, we re-examined the linear mixed models, clarified their specification and diagnostic procedures, and updated the results and interpretation where necessary.

Additional Editor Comments:

Please specify the residual covariance structure, estimation method, and method used to calculate denominator degrees of freedom in the linear mixed models. Clarify how repeated observations within each viewing session and across the two sessions were accounted for, and briefly report how model assumptions were assessed. If these checks identify any issues affecting inference, please revise the analyses and corresponding results accordingly.

Response

Thank you for this comment. We have revised the Statistical analysis section to specify the model structures, estimation method, denominator degrees-of-freedom method, and diagnostic procedures.

All models included fixed effects for video condition, categorical measurement time, and their interaction. Participant-specific random intercepts were included to account for dependence between observations from the same participant across the two viewing sessions. Additional session-specific random intercepts nested within participants were included for HR, LF/HF, HF, cortisol, amylase, and CgA. For LF and VLF, the session-specific random intercepts were omitted after initial fits yielded zero session-level variance estimates and estimation warnings.

Within each participant–session combination, a heterogeneous first-order autoregressive [ARH(1)] residual covariance structure was specified for HR and the HRV indices. This structure allows time-specific residual variances and correlations that depend on the separation between measurement times. A diagonal residual covariance structure allowing time-specific residual variances was specified for the salivary markers; dependence within and across sessions was additionally represented by the session-specific and participant-specific random intercepts, respectively. All models were fitted using restricted maximum likelihood, with denominator degrees of freedom calculated using the Satterthwaite approximation.

We checked convergence and covariance parameter estimates and visually examined residual histograms, Q–Q plots, residual-versus-fitted plots, and residual distributions by measurement time and video condition. These checks identified departures from normality and several large residuals for some outcomes. The corresponding observations were verified and retained, and the analyses were performed on the original measurement scale. We have explicitly acknowledged in the Limitations that the robustness of the model-based estimates, confidence intervals, and significance tests to these departures was not established.

Following this re-examination, we updated the fixed-effect results in Table 3, the estimated condition differences and confidence intervals in Table 4, and the between-condition differences in post-minus-pre changes in S3 Table. The Abstract, Results, Discussion, and Conclusions have been revised consistently with these results. In particular, the revised manuscript now reports a significant condition × time interaction for HR, but not for the other physiological indicators, and a significant average condition difference only for LF. Non-significant findings are not interpreted as evidence of equivalent responses between conditions.

Revised in: Abstract (pp. 2–3, lines 37–41), Materials and Methods (p10, lines 173-192); Results (pp. 17–18, lines 268–297); Table 3 (pp14-15), Table 4 (p16), Discussion (pp19-20, lines 317-337, p23 lines 407-410); Limitations of the Study (p26, lines 465-477), Conclusions (p27, lines 499-504) and Supporting Information (S3 Table).

Thank you for the opportunity to clarify these methodological details.

Sincerely,

Hiroki Matsui, Ph.D.

Department of Laboratory Sciences,

Gunma University Graduate School of Health Sciences
