# Supplementary material for: Cognitive and physiological responses to prolonged video viewing: Activities of the autonomic nervous system and hypothalamo-pituitary-adrenal axis in relation to video interest
Source: PLoS One. 2026 Nov 12;21(11):e0362672. doi: 10.1371/journal.pone.0362672 (PMC13759085; doi:10.1371/journal.pone.0362672)
Supplement: Peer review history

# Author comment: Author response to Decision Letter 0

**DOI:** 10.1371/journal.pone.0362672.r001

1 Dec 2025

Attachment Submitted filename: Response to Reviewers.docx

# Peer review: Decision Letter 0

**DOI:** 10.1371/journal.pone.0362672.r002

**Authors:** Yoko Hasegawa

20 Feb 2026

Dear Dr. Matsui,

Thank you for submitting your manuscript to PLOS ONE. After careful consideration, we feel that it has merit but does not fully meet PLOS ONE’s publication criteria as it currently stands. Therefore, we invite you to submit a revised version of the manuscript that addresses the points raised during the review process.

Please submit your revised manuscript by Apr 06 2026 11:59PM. If you will need more time than this to complete your revisions, please reply to this message or contact the journal office at plosone@plos.org. When you're ready to submit your revision, log on to https://www.editorialmanager.com/pone/ and select the 'Submissions Needing Revision' folder to locate your manuscript file.

- A letter that responds to each point raised by the academic editor and reviewer(s). You should upload this letter as a separate file labeled 'Response to Reviewers'.
- A marked-up copy of your manuscript that highlights changes made to the original version. You should upload this as a separate file labeled 'Revised Manuscript with Track Changes'.
- An unmarked version of your revised paper without tracked changes. You should upload this as a separate file labeled 'Manuscript'.

If you would like to make changes to your financial disclosure, please include your updated statement in your cover letter. Guidelines for resubmitting your figure files are available below the reviewer comments at the end of this letter.

If applicable, we recommend that you deposit your laboratory protocols in protocols.io to enhance the reproducibility of your results. Protocols.io assigns your protocol its own identifier (DOI) so that it can be cited independently in the future. For instructions see: https://journals.plos.org/plosone/s/submission-guidelines#loc-laboratory-protocols. Additionally, PLOS ONE offers an option for publishing peer-reviewed Lab Protocol articles, which describe protocols hosted on protocols.io. Read more information on sharing protocols at https://plos.org/protocols?utm_medium=editorial-email&utm_source=authorletters&utm_campaign=protocols.

We look forward to receiving your revised manuscript.

Kind regards,

Yoko Hasegawa

Academic Editor

PLOS One

**Journal Requirements:**

When submitting your revision, we need you to address these additional requirements.

1. Please ensure that your manuscript meets PLOS ONE's style requirements, including those for file naming. The PLOS ONE style templates can be found at

https://journals.plos.org/plosone/s/file?id=wjVg/PLOSOne_formatting_sample_main_body.pdf and

https://journals.plos.org/plosone/s/file?id=ba62/PLOSOne_formatting_sample_title_authors_affiliations.pdf

2. If the reviewer comments include a recommendation to cite specific previously published works, please review and evaluate these publications to determine whether they are relevant and should be cited. There is no requirement to cite these works unless the editor has indicated otherwise.

**Additional Editor Comments:**

This study examines how prolonged video watching affects the autonomic nervous system and saliva‑based stress markers, comparing “interesting” versus “boring” content in a crossover design. The topic is timely and socially relevant, especially given the growing screen exposure in work and education. The use of both heart rate variability indices (LF/HF, HF, VLF) and salivary biomarkers (cortisol, amylase, chromogranin A) gives the study good physiological depth. However, the paper remains conceptually unclear in some areas, and the analysis and interpretation could be strengthened. I recommend a major revision to make the design, results, and conclusions more coherent.

1. Definition of “Stress”

The paper discusses “stress” during long video watching, but the data (lower HR, higher HF, lower cortisol) point more toward relaxation than stress. It is not clear whether the study actually measures stress, cognitive load, or mental engagement.

Suggestion: Clarify the conceptual focus—are you studying stress, attention, or emotional response? Add a concise explanation linking your HRV and hormonal findings to the relevant physiological systems (HPA and SAM axes).

2. Definition of “Interest”

Using comedy clips versus boring lectures is a reasonable approach, but these differ not only in “interest” but also in emotion, arousal, and cognitive demand.

Suggestion: Explain why this contrast was chosen to represent “interest.” Discuss possible confounding factors and suggest that future work include measures such as engagement scales or cognitive load questionnaires to better isolate the intended variable.

3. Statistical Analysis

The analysis combines t‑tests, Wilcoxon tests, ANOVAs, and AUCs, yet the data are repeated measures. Mixed‑effects models would handle within‑subject variability more appropriately. Consistency in normality testing, correction for multiple comparisons, and interpretation of effect sizes is also needed.

Suggestion: Reanalyze using a mixed‑model framework, or justify your current methods clearly. Provide confidence intervals and interpret effect sizes in physiological terms.

4. Timing of Measures

HRV reflects rapid autonomic fluctuations, while salivary cortisol and related markers respond more slowly. This timing mismatch may explain weak correlations between subjective and physiological outcomes.

Suggestion: Add a short discussion about the different time courses of these measures and how they may affect interpretation.

5. Sample Size and Sex Balance

The sample size (n = 26) is small and sex distribution is uneven, which limits conclusions on sex‑related differences.

Suggestion: State that these analyses are exploratory and emphasize the need for larger, balanced samples in future studies.

6. Additional Considerations

Clarify how LF/HF is interpreted, as its validity is debated. If RR interval data are available, include RMSSD. Briefly explain why VLF was analyzed and its possible physiological meaning. Add a short note relating your experimental setup to real‑life screen exposure contexts, and consider showing correlations between subjective and physiological indicators.

[Note: HTML markup is below. Please do not edit.]

Reviewers' comments:

Reviewer's Responses to Questions

**Comments to the Author**

1. Is the manuscript technically sound, and do the data support the conclusions?

Reviewer #1: Yes

**********

2. Has the statistical analysis been performed appropriately and rigorously?

Reviewer #1: Yes

**********

3. Have the authors made all data underlying the findings in their manuscript fully available?

The PLOS Data policy

Reviewer #1: Yes

**********

4. Is the manuscript presented in an intelligible fashion and written in standard English?

Reviewer #1: Yes

**********

Reviewer #1: The manuscript addresses a common and widespread problem associated with watching prolonged video viewing. By investigating the effects of watching a prolonged video and measuring the activities of the stress axes, they have made a good contribution to the science. Moreover, by making the corrections suggested by the previous reviewer, the manuscript has been improved enormously.

My specific comments are as follows:

-They have measured the activities of autonomous nervous system (ANS) by HRV and CgA and hypothalamo-pituitary-adrenal axis (HPA) by salivary cortisol. However, in the title they have mentioned “physical and mental stress”. From my point of view, it is very much difficult to make such discrimination (i.e. physical and mental) by measuring activities of these two systems (ANS and HPA). To me, the title should change by avoiding “physical and mental” terms, instead ANS and HPA might be incorporated. Moreover the terms “An analysis of..” should be avoided as the study itself is an analysis. Therefore, my suggestion is that the title should be something like: “Activities of the Autonomic nervous system and hypothalmo-pituitary-adrenal axes following viewing (or watching) a prolonged video”. However, this still does not reflect the idea behind the purpose of the study as the participants watched either “high-interest” or “low-interest” video. This is an important side of the study and must be incorporated to the title, purpose, results and conclusions parts. What I mean is that the participants were compared in terms of whether they watch something they like or not. Therefore, this important information should be incorporated to every part of the study.

-In the results section of the abstract, it has been mentioned that there were some differences between the groups but most of them did not reach statistical difference. In modern scientific approach, if there is no statistical difference, then, it cannot be said that there were differences. In other words, they should be accepted as “indifferent”. Therefore, I suggest that the authors make necessary corrections accordingly, wherever necessary.

-In the conclusion section, conclusions are not tightly connected to the data obtained from the current study, rather a wider and inappropriate conclusions are made. Instead, the conclusion should include ANS and HPA changes. Did both increase, decrease or one increased the other is unchanged etc. Afterwards making a generalization saying that this suggest that ANS does not change but salivary cortisol decreases irrespective interest in video. Something like: “Although ANS does not change, HPA activity decreases with video watching irrespective of level of interest in video. The decrease in cortisol level might be important as it is a hormone protecting against harmful effect of stress. Therefore, new studies are warranted to explore the effects of video watching on health in long-term.” Moreover, it would be good if the authors were able to add a word to title to include “concentration, interest, comprehension and fatigue” (probably something like “cognitive”)

-Please correct the last sentence of the results paragraph of the abstract where cortisol is written two times. The last "cortisol" mentioned at the end of the sentence must be CgA.

-Please make a firmer conclusion where the reader can understand which stress axis is affected in which way.

-In terms of presentation of the tables, there are some sort of confusions for the readership. In Table 1, each parameter is compared for high interest vs low interest, and then, the data are presented separately for men and women. As the number of men and women are low, there is no need for such way presentation. For example, in Table 1, P values are all significant and this trend has not changed if the men and women are analyzed separately. Therefore, for me, there is no need to represent data for men and women. Rather, this could be mentioned in text, saying that the trend was similar for men and women. For HRV analyses, again, separate comparisons for men and women does not seem to be necessary. For Table 3, exactly the same trend was observed for men and women for the cortisol, amylase and CgA levels and therefore there is no need to present them in the table. Because, rather than helping the readers, men and women separate comparisons confuses the readership.

-In the discussion part, a sentence describing “what has been done” and “what has been found” must be evaluated at the commencement of discussion. In that respect, a special emphasis must be given to watching “interested video” or “uninterested video”. In this regards, starting the discussion section with “subjective scores” (neither meaningful nor readily related to the purpose of the study) is not a good way of presentation. Rather, a sentence like this could be preferred “In this study, evaluating activities of stress axes (namely ANS and HPA) in response to watching a high and low interest video, we observed that ANS does not change but HPA activity decreases by watching a video irrespective of interest in watching.” After summarizing study in this way (or similar way), the discussion might be followed easily by the readership. After starting discussion, I would recommend subtitles like 1. Video watching did not change ANS as revealed by HRV, amylase and CgA, 2. Video watching decreased cortisol levels irrespective of interest in video, 3. Watching a video increased fatigue and decreased concentration, interest and comprehension when the video was low interest etc.

-Although the authors have not included the time-domain parameters of the HRV, as also suggested by the previous referee, it is understandable from the authors explanation that the device they were using did not calculate these parameters. There is no problem with that but in fact, time-domain parameters are easier to calculate compared to frequency domain parameters like LF, HF and VLF. From my opinion, the number of parameters studied are already quite sufficient to draw scientific conclusions.

**********

what does this mean?). If published, this will include your full peer review and any attached files.

If you choose “no”, your identity will remain anonymous but your review may still be made public.

**Do you want your identity to be public for this peer review?**  For information about this choice, including consent withdrawal, please see our Privacy Policy

Reviewer #1: **Yes:**  Prof. Dr. Sedat YILDIZ

**********

[NOTE: If reviewer comments were submitted as an attachment file, they will be attached to this email and accessible via the submission site. Please log into your account, locate the manuscript record, and check for the action link "View Attachments". If this link does not appear, there are no attachment files.]

To ensure your figures meet our technical requirements, please review our figure guidelines: https://journals.plos.org/plosone/s/figures

You may also use PLOS’s free figure tool, NAAS, to help you prepare publication quality figures: https://journals.plos.org/plosone/s/figures#loc-tools-for-figure-preparation.

NAAS will assess whether your figures meet our technical requirements by comparing each figure against our figure specifications.

# Author comment: Author response to Decision Letter 1

**DOI:** 10.1371/journal.pone.0362672.r003

14 Mar 2026

We sincerely appreciate your thoughtful and constructive comments on the manuscript titled “Cognitive and Physiological Responses to Prolonged Video Viewing: Autonomic Nervous System and Hypothalamo-Pituitary-Adrenal Axis Activity Related to Interest in Videos” (formerly titled: Effects of Prolonged Video Viewing on Physical and Mental Stress: Analysis of Autonomic Nervous Activity and Salivary Stress Markers).

We have carefully considered all points raised by the editor and reviewers. We believe these revisions have significantly improved the manuscript. Our responses to each comment are provided below.

Responses to Academic Editor

Comment 1. Definition of “Stress”

The data (lower HR, higher HF, lower cortisol) point more toward relaxation than stress. Clarify the conceptual focus.

Response:

We appreciate this important point. We agree that the original wording overly emphasized “stress” and did not sufficiently distinguish between physiological stress, cognitive engagement, and subjective experience. Therefore, we clarified that while this study evaluates autonomic nervous system (ANS) and hypothalamo-pituitary-adrenal (HPA) axis-related physiological responses during prolonged video viewing, subjective assessments reflect cognitive and experiential aspects such as concentration, comprehension, interest, and fatigue. We also revised the Discussion section to interpret the decreases in heart rate and the LF/HF ratio, together with the increase in HF and the reduction in salivary cortisol, as findings consistent with relative relaxation rather than increased physiological stress. Furthermore, we added an explanation in the introduction regarding the distinct roles and temporal dynamics of the HPA axis and the sympathetic-adrenal medullary (SAM) system.

Revised In: Title (p 1, lines 1-3), Abstract (p 2-3, lines 19–41), Introduction (p 4, lines 50–52; p5-6, lines 64-86), Discussion (p 14-18, lines 263–317), and Conclusions (p 21-22, lines 387–392).

Comment 2. Definition of “Interest”

Using comedy vs. lectures involves confounding factors (emotion, arousal). Explain the choice and discuss limitations.

Response:

As you pointed out, we have clarified the reasons for selecting comedy videos and lecture videos. The rationale for using contrast videos was to maximize differences in subjective engagement under a controlled, extended viewing protocol. Simultaneously, we explicitly stated that these stimuli differ not only in interest but also in emotional valence, arousal, and cognitive load. Thus, we did not isolate “interest” as the sole causal factor. We added these points to both the Discussion and Limitation sections and propose incorporating standardized measurement methods, such as cognitive load and immersion scales, into future analyses.

Revised In: Discussion (p 19, lines 325–333), and Limitations (p 20, lines 353–363).

Comment 3. Statistical Analysis

Use mixed-effects models to handle within-subject variability.

Response:

Thank you for your suggestion. In response, we reanalyzed the repeated-measures physiological outcomes using a linear mixed-effects model. We included interest in the video and time as fixed effects, along with their interaction, and accounted for within-subjects correlation by including a subject-specific random intercept. We also added post-hoc comparisons based on the fitted model, reporting estimated mean differences and 95% confidence intervals. To assess model fit, we included marginal R² and conditional R² values. The results are presented in Table 3 and the Results section, with interpretations discussed in the Discussion.

Revised In: Methods (p 9, lines 154–160), Results (p 11, lines 199–201; p 14-15, lines 222–249), Discussion (p 16-18, lines 271–317), and Table 3.

Comment 4. Timing of Measures

Discuss the timing mismatch between HRV and salivary cortisol.

Response:

We agree with this perspective and have added clarification to the Discussion section (Section 2). While HRV reflects relatively rapid fluctuations in the autonomic nervous system, salivary cortisol changes more gradually and may reflect physiological responses to psychological events that occurred earlier. This temporal lag contributes to the discrepancy between subjective scores and endocrine markers.

Revised In: Discussion (p 18, lines 311–317) and Limitations (p 21, lines 374–381).

Comment 5. Sample Size and Sex Balance

State that sex analyses are exploratory due to small sample size.

Response:

This paper explicitly states that, due to the small sample size and uneven sex distribution, sex-related analyses should be interpreted cautiously as exploratory. Furthermore, in the “Discussion” and “Limitations” sections, we explicitly stated that our analysis of sex differences is preliminary and exploratory. As shown in Fig. 2–4, we removed the sex-specific figures and presented only the results for the overall sample.

Revised In: Results (p 15, lines 250–251), Discussion (p 19, lines 336–341), Limitations (p 20-21, lines 364–373), and Fig. 2-4.

Comment 6. Additional Considerations (LF/HF, VLF, RMSSD)

Response:

We have addressed the points you raised as follows. First, we supplemented the Discussion section to note that while LF/HF is widely used, its status as a pure indicator of sympathetic activity remains debatable. Second, we added a concise explanation regarding VLF and its physiological significance during prolonged viewing. Third, we added interpretation linking the 90-minute viewing protocol to real-life scenarios such as university lectures and remote meetings. Fourth, we added a correlation analysis between subjective and physiological indicators, summarizing the results in S2 Table.

Regarding RMSSD, since this study primarily used device outputs centered on frequency-domain HRV measurements, the revised manuscript maintains the frequency-domain framework. To clearly indicate this to readers, we added the following sentence to the method section: Time-domain HRV parameters, including RMSSD, were not analyzed because they were not provided by the measurement device used in this study.

Revised In: Methods (p 7-8, lines 127–129), Results (p 15, lines 253–261), Discussion (p 17, lines 289–295; p 19, lines 342-350), Limitations (p 19-20, lines 342–350), and Supporting Information (Table S2).

Responses to Reviewer #1

Comment 1. Title: Avoid “physical and mental stress” and incorporate ANS/HPA and video interest.

Response:

We appreciate this valuable suggestion. We agree that the previous title overemphasized what could be inferred from the measured physiological indicators. Therefore, we have revised the title to focus on the ANS and HPA axis activity and to reflect the role of interest in the video. We have also removed the phrase “An analysis of,” as you suggested.

Revised In: Title (p 1, lines 1–3).

Comment 2. The purpose, results, and conclusion should better reflect the high-interest versus low-interest design.

Response:

We revised the abstract, introduction, discussion, and conclusion to ensure the distinction between high-interest and low-interest videos is more clearly described throughout the manuscript. Simultaneously, we clarified that although subjective reactions varied with video interest level, no significant interaction between interest level and time was observed for physiological reactions.

Revised In: Title (p 1, lines 1-3), Abstract (p 2-3, lines 19–41), Introduction (p 4, lines 50–52; p5-6, lines 64-86), Discussion (p 14-18, lines 263–317; p 19, lines 325–333), and Conclusions (p 21-22, lines 387–392).

3. Comment 3. The conclusion was too broad and should more directly reflect ANS and HPA findings

Response:

As you pointed out, we revised the conclusion section to directly reflect the data. The revised conclusion states that while interest in the video influenced subjective cognitive evaluations, physiological responses were generally similar across conditions. Salivary cortisol decreased over time in both conditions, indicating reduced HPA axis activity. Meanwhile, HRV indicators changed over time, but no clear dependence on interest levels was observed.

Revised In: Conclusions (p 21-22, lines 384–392).

Comment 4. Abstract typo: cortisol was written twice, and the last term should be CgA

Response:

Thank you for pointing out this error. We corrected the Abstract accordingly.

Revised In: Abstract (p 2, lines 35–36).

Comment 5. Please make the conclusion firmer regarding which stress axis changed and in what way

Response:

As you pointed out, to more clearly indicate the direction of the physiological findings, we revised both the opening section of the discussion and the conclusion section. Specifically, we explicitly stated that while no clear interest-dependent changes were observed in autonomic nervous system/adrenal cortex system (ANS/SAM) markers, the HPA axis marker, salivary cortisol, decreased over time under both viewing conditions.

Revised In: Discussion (p. 16, lines 264–267) and Conclusions (p 21-22, lines 384–392).

Comment 6. The tables are confusing, and a sex-separated presentation is unnecessary

Response:

We agree with this practical suggestion. We simplified the main tables and focused the study's main presentation on intra-subject comparisons and mixed-model results (Tables 1-3). Unnecessary sex-stratified presentations were removed. Instead, sex-related analyses were concisely summarized in the main text, with detailed results retained in supplementary information (Table S1). Additionally, sex-specific results in Figures 2–4 have been removed, and only the overall sample results are presented.

Revised In: Tables 1–3, Results (p 11-15, lines 192–261), Fig. 2-4, and Supporting Information (Table S1).

Comment 7. The Discussion should begin by stating what was done and what was found

Response:

As you pointed out, we revised the opening paragraph of the discussion section. We restructured it to first concisely summarize the study design and key findings, then transition to the interpretation by theme. We also reorganized the discussion's structure using clearer subheadings.

Revised In: Discussion (p. 16-19, lines 264–333).

Comment 8: Time-domain HRV parameters were not included.

Response:

Thank you for your comment. Since this study was conducted using device outputs centered on frequency-domain HRV indices, we adopted LF/HF, HF, and VLF as the primary HRV indices. Furthermore, in the Discussion section, we clarified that these indices are sufficient to support physiological interpretations. Regarding RMSSD, to indicate that it was not output by the device used in this study, we added the following sentence to the Methods section:

Time-domain HRV parameters, including RMSSD, were not analyzed because they were not provided by the measurement device used in this study.

Revised In: Methods (p 7-8, lines 127–129), and Discussion (p. 16-17, lines 269–301).

We hope that these revisions have adequately addressed the Editor’s and Reviewer’s concerns, and we are grateful for the thoughtful comments, which have helped us improve the clarity and rigor of the manuscript.

Attachment Submitted filename: Response to Reviewers 20250313.docx

# Peer review: Decision Letter 1

**DOI:** 10.1371/journal.pone.0362672.r004

**Authors:** Yoko Hasegawa

13 Apr 2026

Dear Dr. Matsui,

Thank you for submitting your manuscript to PLOS ONE. After careful consideration, we feel that it has merit but does not fully meet PLOS ONE’s publication criteria as it currently stands. Therefore, we invite you to submit a revised version of the manuscript that addresses the points raised during the review process.

Please submit your revised manuscript by May 28 2026 11:59PM. If you will need more time than this to complete your revisions, please reply to this message or contact the journal office at plosone@plos.org. When you're ready to submit your revision, log on to https://www.editorialmanager.com/pone/ and select the 'Submissions Needing Revision' folder to locate your manuscript file.

- A letter that responds to each point raised by the academic editor and reviewer(s). You should upload this letter as a separate file labeled 'Response to Reviewers'.
- A marked-up copy of your manuscript that highlights changes made to the original version. You should upload this as a separate file labeled 'Revised Manuscript with Track Changes'.
- An unmarked version of your revised paper without tracked changes. You should upload this as a separate file labeled 'Manuscript'.

If you would like to make changes to your financial disclosure, please include your updated statement in your cover letter. Guidelines for resubmitting your figure files are available below the reviewer comments at the end of this letter.

If applicable, we recommend that you deposit your laboratory protocols in protocols.io to enhance the reproducibility of your results. Protocols.io assigns your protocol its own identifier (DOI) so that it can be cited independently in the future. For instructions see: https://journals.plos.org/plosone/s/submission-guidelines#loc-laboratory-protocols. Additionally, PLOS ONE offers an option for publishing peer-reviewed Lab Protocol articles, which describe protocols hosted on protocols.io. Read more information on sharing protocols at https://plos.org/protocols?utm_medium=editorial-email&utm_source=authorletters&utm_campaign=protocols.

As the corresponding author, your ORCID iD is verified in the submission system and will appear in the published article. PLOS supports the use of ORCID, and we encourage all coauthors to register for an ORCID iD and use it as well. Please encourage your coauthors to verify their ORCID iD within the submission system before final acceptance, as unverified ORCID iDs will not appear in the published article. *Only*  the individual author can complete the verification step; PLOS staff *cannot*  verify ORCID iDs on behalf of authors.

We look forward to receiving your revised manuscript.

Kind regards,

Yoko Hasegawa

Academic Editor

PLOS One

Journal Requirements:

If the reviewer comments include a recommendation to cite specific previously published works, please review and evaluate these publications to determine whether they are relevant and should be cited. There is no requirement to cite these works unless the editor has indicated otherwise.

**Additional Editor Comments:**

I have carefully reviewed the manuscript. The study addresses a relevant and timely topic and employs a multimodal approach that combines subjective assessments with physiological measures, including autonomic and hypothalamo-pituitary-adrenal axis activity.

However, despite its potential, the manuscript contains several significant methodological and interpretative issues that limit the strength and clarity of its conclusions. In particular, concerns regarding the experimental design, adequacy of control conditions, sensitivity and interpretation of physiological indices, and statistical reporting need to be addressed. Furthermore, several important points require clarification to ensure the validity and reproducibility of the findings.

Given these considerations, I believe that the manuscript requires major revision before it can be considered for publication.

[Note: HTML markup is below. Please do not edit.]

Reviewers' comments:

Reviewer's Responses to Questions

**Comments to the Author**

Reviewer #2: (No Response)

Reviewer #3: (No Response)

**********

2. Is the manuscript technically sound, and do the data support the conclusions?

Reviewer #2: Partly

Reviewer #3: Partly

**********

3. Has the statistical analysis been performed appropriately and rigorously?

Reviewer #2: Yes

Reviewer #3: Yes

**********

4. Have the authors made all data underlying the findings in their manuscript fully available?

The PLOS Data policy

Reviewer #2: Yes

Reviewer #3: Yes

**********

5. Is the manuscript presented in an intelligible fashion and written in standard English?

Reviewer #2: Yes

Reviewer #3: Yes

**********

Reviewer #2: The study investigated the effects of high-interest (humorous video) and low-interest (educational video) content on stress parameters. The topic of the study is interesting. The manuscript has improved after the necessary revisions and appears stronger. However, there are still methodological concerns in the study.

1- In the abstract, the terms ‘high-interest’ and ‘low-interest’ videos are used. However, the content of these videos is not specified. For better clarity, the type of videos should be clearly stated in the abstract. For example, high-interest video (humorous video) should be indicated.

2- The revised abstract is better written than the previous version. The earlier abstract clearly required complete rewriting.

3- In this type of study, to minimize the temporal effect, it would be more appropriate to start with low-interest videos in some participants and high-interest videos in others. It is understood that randomizing the video order was intended to eliminate this temporal difference. However, this is not clearly shown in Figure 1.

“4- In the discussion, it is stated that the LF/HF parameter is controversial as a marker of sympathetic activity. This is correct; however, when only the LF/HF ratio is considered, it is important to determine whether the change is due to LF or HF components. Therefore, LF and HF parameters should also be evaluated separately. After mentioning that it is controversial, it would be more appropriate to interpret your findings based on the changes in LF and HF and explain whether they reflect a relaxed state.

5- In the discussion, the decrease in cortisol levels is explained only in terms of video duration. This suggests a methodological weakness, because the aim of the study was not to investigate the effect of video duration.

Reviewer #3: The study titled “Cognitive and Physiological Responses to Prolonged Video Viewing: Activities of the Autonomic Nervous System and Hypothalamo-Pituitary-Adrenal Axis in Relation to Video Interest” addresses a timely and relevant topic, particularly in light of the increasing prevalence of online learning and prolonged screen exposure. The integration of subjective cognitive evaluations with objective physiological measures, including autonomic and Hypothalamic-Pituitary-Adrenal Axis activity, represents a valuable and commendable approach. The attempt to explore potential dissociations between perceived experience and physiological responses is especially noteworthy.

However, despite its conceptual strengths, the study presents several methodological and interpretative issues that require clarification and improvement. Certain aspects of the experimental design, measurement sensitivity, and data interpretation remain insufficiently addressed, limiting the strength of the conclusions. Therefore, while the study is interesting and potentially valuable, several points need to be revised, clarified, and further elaborated to enhance its scientific rigor and overall contribution.

1. The sample size is insufficient: The study includes only 26 participants, which limits statistical power and reduces the generalizability of the findings.

2. Limited population diversity: All participants are university students within a narrow age range (21–24 years), limiting the applicability of the findings to broader and more diverse populations.

3. The experimental duration is excessively long: A 90-minute viewing period may induce mental fatigue and reduced attention, confounding the effects of the independent variable.

4. Time effects are not adequately controlled: It is unclear whether the observed physiological changes are due to video content or simply prolonged exposure over time.

5. Lack of a proper control condition: The absence of control conditions (e.g., passive rest, quiet sitting, or alternative cognitive tasks) weakens causal interpretation.

6. Limited sensitivity of physiological measures: Similar ANS and HPA responses in both high- and low-interest conditions raise concerns about the sensitivity of the measurements.

7. Oversimplified interpretation of HRV parameters: The study interprets LF/HF ratio and HF power as indicators of autonomic balance without acknowledging ongoing debates about their validity.

8. Insufficient endocrine assessment: Only cortisol, amylase, and chromogranin A were measured, which provides a limited evaluation of the Hypothalamic-Pituitary-Adrenal Axis.

9. Circadian rhythm of cortisol not addressed: Cortisol levels naturally fluctuate throughout the day, yet timing and control for circadian variation are not clearly discussed.

10. Weak interpretation of subjective–objective dissociation: The conclusion that subjective experience and physiological responses are dissociated is not sufficiently supported, as alternative explanations are not ruled out.

11. Potential bias in subjective measures: Self-reported ratings are vulnerable to social desirability and expectation bias.

12. Limited statistical reporting: Effect sizes and power analysis are not reported, limiting the interpretation of the findings.

13. Interpretation despite non-significant interaction effects: Conclusions about content-related differences are made despite the absence of significant interaction effects.

14. Timing of biomarker collection may be suboptimal: Salivary sampling points may not adequately capture rapid or transient physiological changes.

15. Single-session study design: The use of a single session increases inter-individual variability and reduces reliability.

16. Lack of central nervous system measures: The absence of neurophysiological methods (e.g., EEG or neuroimaging) limits interpretation at the brain level.

**********

what does this mean?). If published, this will include your full peer review and any attached files.

If you choose “no”, your identity will remain anonymous but your review may still be made public.

**Do you want your identity to be public for this peer review?**  For information about this choice, including consent withdrawal, please see our Privacy Policy

Reviewer #2: No

Reviewer #3: No

**********

[NOTE: If reviewer comments were submitted as an attachment file, they will be attached to this email and accessible via the submission site. Please log into your account, locate the manuscript record, and check for the action link "View Attachments". If this link does not appear, there are no attachment files.]

To ensure your figures meet our technical requirements, please review our figure guidelines: https://journals.plos.org/plosone/s/figures

You may also use PLOS’s free figure tool, NAAS, to help you prepare publication quality figures: https://journals.plos.org/plosone/s/figures#loc-tools-for-figure-preparation.

NAAS will assess whether your figures meet our technical requirements by comparing each figure against our figure specifications.

Attachment Submitted filename: Reviewer Comment.docx

# Author comment: Author response to Decision Letter 2

**DOI:** 10.1371/journal.pone.0362672.r005

10 Oct 2026

Thank you for your careful evaluation of our manuscript and for your constructive comments. We revised the manuscript to clarify the study design, improve statistical reporting, and interpret the physiological findings more cautiously.

The principal revisions include separate evaluation of LF and HF power, reporting of model-estimated differences and their 95% confidence intervals, clarification of the randomized viewing order and saliva collection times, and expanded discussion of methodological limitations. We have also distinguished average between-condition differences from differences in temporal trajectories and revised statements that could imply causal effects or equivalent responses despite non-significant interactions.

We have carefully considered all points raised by the editor and reviewers. We believe these revisions have significantly improved the manuscript.

Our point-by-point responses are provided below.

Responses to Reviewer #2

Comment 1

In the abstract, the terms ‘high-interest’ and ‘low-interest’ videos are used. However, the content of these videos is not specified. For better clarity, the type of videos should be clearly stated in the abstract. For example, high-interest video (humorous video) should be indicated.

Response:

Thank you for this suggestion. We have specified the video content in the Abstract as high-interest (comedy) and low-interest (lecture) videos. This terminology is consistent with the description of the video conditions in the Methods.

Revised in: Abstract (p. 2, lines 24–25).

Comment 2

The revised abstract is better written than the previous version. The earlier abstract clearly required complete rewriting.

Response:

Thank you for your positive assessment of the revised Abstract. We further refined it to specify the video content, include LF power among the physiological measures, and distinguish observed time effects and average between-condition differences from non-significant interest × time interactions.

Revised in: Abstract (pp. 2–3, lines 24–41).

Comment 3

In this type of study, to minimize the temporal effect, it would be more appropriate to start with low-interest videos in some participants and high-interest videos in others. It is understood that randomizing the video order was intended to eliminate this temporal difference. However, this is not clearly shown in Figure 1.

Response:

We agree that the viewing order required clearer explanation. Each participant completed both video conditions on separate days, and the viewing order was randomized across participants. Some participants viewed the high-interest video first, whereas others viewed the low-interest video first.

We have revised the Methods and Figure 1 to explicitly describe these two sequences. We also clarified that we conducted the sessions at the same time of day for each participant. We intended randomization to reduce systematic order and period effects; however, we now acknowledge that it does not rule out order effects or day-to-day influences.

Revised in: Methods (p. 7, lines 112–114), Fig 1 and Fig 1 legend (p. 7, lines 118–125), Limitations (p. 26, lines 467–470).

Comment 4

In the discussion, it is stated that the LF/HF parameter is controversial as a marker of sympathetic activity. This is correct; however, when only the LF/HF ratio is considered, it is important to determine whether the change is due to LF or HF components. Therefore, LF and HF parameters should also be evaluated separately. After mentioning that it is controversial, it would be more appropriate to interpret your findings based on the changes in LF and HF and explain whether they reflect a relaxed state.

Response:

Thank you for this important comment. We have added the LF analysis and now report LF and HF separately, together with LF/HF.

Both LF and HF showed significant main effects of time and increased during viewing. Thus, LF/HF decreased alongside increases in both components rather than a decrease in LF. We have revised the Discussion to explain that this pattern may reflect relative changes in the two components.

Across measurement time points, LF was higher in the high-interest condition, whereas HF showed no significant main effect of interest. The higher LF/HF ratio in the high-interest condition was therefore consistent with the observed LF difference, although the relative contributions of LF and HF to the ratio were not directly quantified. Furthermore, no significant interaction was observed between the variables of interest and time for either LF or HF.

We also noted that LF/HF is not a specific measure of sympathetic activity and that HF is influenced by respiration and prevailing heart rate. The decrease in heart rate and increase in HF may be consistent with increased cardiac vagal modulation and a more relaxed physiological state, but they do not establish parasympathetic activation or relaxation.

Revised in: Abstract (p. 2, lines 27–34), Introduction (pp. 4-5, lines 54–65), Methods (p. 8, lines 130–138), Results (p. 16, lines 257–268), Table 2 (p. 13), Table 3 (p. 14), Table 4 (p. 15), Fig 3, Discussion (pp. 19–20, lines 329–345) and Supporting Information (S1-3 Table).

Comment 5

In the discussion, the decrease in cortisol levels is explained only in terms of video duration. This suggests a methodological weakness, because the aim of the study was not to investigate the effect of video duration.

Response:

We agree and have revised the interpretation. Because viewing duration was fixed at 90 minutes, the present study cannot determine the effect of varying video duration or establish that duration has a greater influence than video content.

The revised Discussion considers several possible contributors to the cortisol decrease, including elapsed time, habituation to the experimental setting, normal diurnal decline, and task context. We explicitly state that the present design cannot distinguish these contributions and that the decrease does not, by itself, establish reduced physiological stress attributable to video viewing.

Revised in: Discussion (p. 21, lines 357–376) and Conclusions (p. 26, lines 478–480).

Responses to Reviewer #3

Comment 1

The sample size is insufficient: The study includes only 26 participants, which limits statistical power and reduces the generalizability of the findings.

Response:

We agree that the sample size limits both statistical power and generalizability. We have explicitly acknowledged these limitations and the possibility that small effects were not detected.

To improve interpretation of the findings, we report model-estimated between-condition differences and their 95% confidence intervals in Table 4 and post-minus-pre viewing changes and their 95% confidence intervals in S3 Table. These estimates describe the magnitude and precision of the reported contrasts, but they do not resolve the limitations of the small sample. We also state that non-significant interactions do not establish equivalent responses between conditions.

Revised in: Methods (p. 10, lines 172–181), Results (p. 12, lines 222–224), Table 4 and its legend (pp. 15–16), Limitations (p. 25, lines 442–454), and Supporting Information (S3 Table).

Comment 2

Limited population diversity: All participants are university students within a narrow age range (21–24 years), limiting the applicability of the findings to broader and more diverse populations.

Response:

As you pointed out, the age range for this initiative is limited. The Limitations now explicitly state that all participants were university students aged 21–24 years and that the findings may not be applicable to other age groups or populations. Accordingly, the conclusions are restricted to the responses observed in the present sample and viewing conditions.

Revised In: Limitations (p. 25, lines 442–444).

Comment 3

The experimental duration is excessively long: A 90-minute viewing period may induce mental fatigue and reduced attention, confounding the effects of the independent variable.

Response:

We selected the 90-minute protocol to reflect prolonged viewing in everyday settings, such as university lectures and feature-length films. Nevertheless, we agree that fatigue and reduced attention may have developed during viewing and contributed to the physiological changes.

We added this limitation and clarified that these contributions cannot be separated from those of video content because we obtained subjective ratings only after viewing. We have also revised the description of the subjective findings to refer to differences between conditions after viewing, rather than implying changes from pre-viewing levels.

Revised in: Discussion (p. 22, lines 383–390) and Limitations (p. 24, lines 434–438).

Comment 4

Time effects are not adequately controlled: It is unclear whether the observed physiological changes are due to video content or simply prolonged exposure over time.

Response:

We agree that a significant main effect of time cannot identify the cause of the physiological changes. We have revised the Discussion to acknowledge that elapsed time, habituation, prolonged seated rest, and, for cortisol, diurnal decline may have contributed.

We also distinguish average between-condition differences from differences in temporal trajectories. No significant interest × time interactions were detected. Furthermore, because the average condition estimates include pre-viewing measurements, we clarify that they do not necessarily represent differences induced by video viewing.

Revised in: Discussion (pp. 18–19, lines 305–312; p. 21, lines 362–376), Limitations (pp. 24–25, lines 438–441), and Conclusions (p. 26, lines 478–480).

Comment 5

Lack of a proper control condition: The absence of control conditions (e.g., passive rest, quiet sitting, or alternative cognitive tasks) weakens causal interpretation.

Response:

Thank you for this important comment. We have explicitly acknowledged the absence of a non-viewing control condition, such as quiet sitting or an alternative cognitive task.

The revised manuscript states that the observed changes cannot be attributed specifically to video viewing or distinguished from changes that might have occurred over the same period without video exposure. We have also revised the interpretation and conclusions to avoid attributing the physiological findings solely to the videos.

Revised in: Introduction (p. 5, lines 83–85), Discussion (p. 19, lines 310–312; p. 21, lines 357–365), Limitations (pp. 24–25, lines 438–441), and Conclusions (p. 26, lines 478–480).

Comment 6

Limited sensitivity of physiological measures: Similar ANS and HPA responses in both high- and low-interest conditions raise concerns about the sensitivity of the measurements.

Response:

We agree that non-significant differences may reflect limited measurement sensitivity, sampling timing, or statistical power. We now discuss these alternative explanations and avoid interpreting the findings as evidence that the physiological responses were equivalent.

The results also warrant distinction between average levels and temporal trajectories: HR, LF/HF, LF, and VLF showed significant average between-condition differences, whereas no physiological indicator showed a significant interest × time interaction. Table 4 provides the corresponding estimates and confidence intervals. For non-significant contrasts, the confidence intervals illustrate the remaining uncertainty rather than establishing the absence of a physiological response.

Revised in: Results (p. 16, lines 252–268; p. 17, lines 269–277), Table 4 and its legend (pp. 15–16), Discussion (p. 20, lines 346–353; pp. 22–23, lines 396–399), and Limitations (p. 25, lines 449–453).

Comment 7

Oversimplified interpretation of HRV parameters: The study interprets LF/HF ratio and HF power as indicators of autonomic balance without acknowledging ongoing debates about their validity.

Response:

Thank you for highlighting this issue. We have revised the Introduction, Methods, and Discussion to acknowledge the limitations of HRV interpretation.

LF/HF is now described as a frequency-domain HRV measure rather than a direct measure of sympathetic activity or sympathovagal balance. We clarify that LF reflects multiple regulatory influences and that HF is associated with cardiac vagal modulation but is also influenced by respiration and prevailing heart rate. We interpret LF and HF separately and in conjunction with heart rate, and we avoid concluding that the observed pattern establishes sympathetic withdrawal, parasympathetic activation, or relaxation.

Revised in: Introduction (pp. 4-5, lines 54–65), Methods (p. 8, lines 133–138), and Discussion (pp. 19–20, lines 329–345).

Comment 8

Insufficient endocrine assessment: Only cortisol, amylase, and chromogranin A were measured, which provides a limited evaluation of the Hypothalamic-Pituitary-Adrenal Axis.

Response:

We agree with that point and have clarified the roles of these markers. HPA axis assessment in this study was limited to salivary cortisol. Amylase and CgA provided complementary autonomic/SAM-related information rather than direct measures of HPA axis activity.

We acknowledge that the absence of additional endocrine measures, such as ACTH, limits characterization of HPA axis regulation.

Revised in: Limitations (p. 26, lines 463–466).

Comment 9

Circadian rhythm of cortisol not addressed: Cortisol levels naturally fluctuate throughout the day, yet timing and control for circadian variation are not clearly discussed.

Response:

We have clarified the sampling schedule and its limitations. Saliva samples were collected between 09:00 and 12:00 or between 13:00 and 17:00. For each participant, the two sessions were conducted at the same time of day on separate days, with corresponding samples collected at matched times.

This procedure was intended to reduce time-of-day confounding in comparisons between conditions. However, we explicitly acknowledge that matching session times does not eliminate natural cortisol decline within each session and that differences in sampling times between participants may contribute to variability. We have added a reference supporting diurnal cortisol decline and included this alternative explanation in the Discussion and Conclusions.

Revised in: Methods (p. 8, lines 141–146), Discussion (p. 21, lines 369–376), and Conclusions (p. 26, lines 478–480).

Comment 10

Weak interpretation of subjective–objective dissociation: The conclusion that subjective experience and physiological responses are dissociated is not sufficiently supported, as alternative explanations are not ruled out.

Response:

We agree with this perspective and have moderated this interpretation. The revised manuscript describes differing patterns of subjective ratings and measured physiological responses rather than an established dissociation.

We explicitly acknowledge that limited statistical power, measurement sensitivity, and differences in response timing may explain the findings. We also recognize that the comedy and lecture videos differed in emotional valence, arousal, and cognitive load, preventing interest from being isolated as a single causal factor.

Revised in: Abstract (p. 3, lines 39–41), Discussion (pp. 22–23, lines 386–402) and Conclusions (p. 26, lines 474–482).

Comment 11

Potential bias in subjective measures: Self-reported ratings are vulnerable to social desirability and expectation bias.

Response:

Thank you for your comment. We have added a statement acknowledging that the subjective ratings may have been influenced by participants’ expectations and social desirability. These ratings are interpreted as self-reported evaluations rather than objective measures of cognitive performance.

Revised in: Limitations (p. 24, lines 437–438).

Comment 12

Limited statistical reporting: Effect sizes and power analysis are not reported, limiting the interpretation of the findings.

Response:

We have expanded the statistical reporting. Table 1 reports effect sizes for the paired comparisons of subjective ratings. Table 3 reports marginal and conditional R² for each mixed model, and the Methods and Discussion clarify that these represent model-level explained variance rather than the magnitude of individual fixed effects.

Table 4 reports model-estimated between-condition differences averaged across measurement time points and their 95% confidence intervals. S3 Table reports post-minus-pre viewing changes averaged across video conditions and their 95% confidence intervals.

No additional power analysis was performed. We explicitly acknowledge this and clarify that the confidence intervals describe the precision of the reported contrasts but do not quantify power to detect interest × time interactions. Therefore, sample-size limitations remain part of the interpretation.

Revised in: Methods (pp. 9–10, lines 168–181), Results (p. 12, lines 222–224), Table 1 (p. 12), Table 3 and its legend (pp. 14–15), Table 4 and its legend (pp. 15–16), Discussion (p. 19, lines 313–316), Limitations (p. 25, lines 444–453), and Supporting Information (S3 Table).

Comment 13

Interpretation despite non-significant interaction effects: Conclusions about content-related differences are made despite the absence of significant interaction effects.

Response:

We agree that condition main effects and condition × time interactions must be interpreted separately. We have revised the Abstract, Results, Discussion, and Conclusions accordingly.

The manuscript now distinguishes the average between-condition differences observed for HR, LF/HF, LF, and VLF from the absence of significant differences in temporal trajectories. We do not interpret the non-significant interactions as evidence of equivalent responses. We also acknowledge that the average condition estimates include pre-viewing measurements and do not necessarily represent differences induced by video viewing.

Revised in: Abstract (pp. 2–3, lines 33–41), Results (p. 16, lines 254–268; p. 17, lines 269–277), Table 4 legend (p. 16, lines 247–250), Discussion (pp. 18–19, lines 305–310), Limitations (p. 25, lines 449–453), and Conclusions (p. 26, lines 474–482).

Comment 14

Timing of biomarker collection may be suboptimal: Salivary sampling points may not adequately capture rapid or transient physiological changes.

Response:

We appreciate this important point. We clarified that we collected saliva immediately before viewing, 45 minutes after viewing began, and immediately after completing the 90-minute video.

The revised Discussion and Limitations acknowledge that this schedule may have missed rapid or transient responses to particular portions of the videos and cannot fully characterize the temporal relationship between salivary markers and HRV. We also discuss the delayed cortisol response relative to rapid autonomic fluctuations.

Revised in: Methods (p. 8, lines 141–143), Discussion (pp. 21–22, lines 376–381), and Limitations (p. 25, lines 455–460).

Comment 15

Single-session study design: The use of a single session increases inter-individual variability and reduces reliability.

Response:

We apologize that the original description did not sufficiently clarify the design. Each participant completed two sessions on separate days, one for each video condition, in randomized order. The main repeated-measures analyses included subject-specific random intercepts to account for within-participant correlation.

Nevertheless, we assessed each condition only once. We therefore agree that within-condition test–retest reliability could not be evaluated. We clarified the two-session design and added this limitation, including potential order and day-to-day influences.

Revised in: Methods (p. 7, lines 110–114), Fig 1 and Fig 1 legend (p. 7, lines 118–125), and Limitations (p. 26, lines 467–470).

Comment 16

Lack of central nervous system measures: The absence of neurophysiological methods (e.g., EEG or neuroimaging) limits interpretation at the brain level.

Response:

We agree with that point. We have added a statement acknowledging that EEG and neuroimaging were not performed and that the peripheral physiological findings do not permit direct conclusions about central nervous system activity.

Revised In: Limitations (p. 26, lines 470–471).

We hope these revisions have adequately addressed the Editor’s and Reviewers’ concerns, and we are grateful for the thoughtful comments, which helped us improve the manuscript's clarity and rigor.

Attachment Submitted filename: Response to Reviewers 20261010.docx

# Editor report: Decision Letter 2

**DOI:** 10.1371/journal.pone.0362672.r006

**Authors:** Yoko Hasegawa

14 Oct 2026

Dear Dr. Matsui,

Thank you for submitting your manuscript to PLOS One. After careful consideration, we feel that it has merit but does not fully meet PLOS One’s publication criteria as it currently stands. Therefore, we invite you to submit a revised version of the manuscript that addresses the points raised during the review process.

Please submit your revised manuscript by Nov 28 2026 11:59PM. If you will need more time than this to complete your revisions, please reply to this message or contact the journal office at plosone@plos.org. When you're ready to submit your revision, log on to https://www.editorialmanager.com/pone/ and select the 'Submissions Needing Revision' folder to locate your manuscript file.

- A letter that responds to each point raised by the academic editor and reviewer(s). You should upload this letter as a separate file labeled 'Response to Reviewers'.
- A marked-up copy of your manuscript that highlights changes made to the original version. You should upload this as a separate file labeled 'Revised Manuscript with Track Changes'.
- An unmarked version of your revised paper without tracked changes. You should upload this as a separate file labeled 'Manuscript'.

If you would like to make changes to your financial disclosure, please include your updated statement in your cover letter. Guidelines for resubmitting your figure files are available below the reviewer comments at the end of this letter.

If applicable, we recommend that you deposit your laboratory protocols in protocols.io to enhance the reproducibility of your results. Protocols.io assigns your protocol its own identifier (DOI) so that it can be cited independently in the future. For instructions see: https://journals.plos.org/plosone/s/submission-guidelines#loc-laboratory-protocols. Additionally, PLOS One offers an option for publishing peer-reviewed Lab Protocol articles, which describe protocols hosted on protocols.io. Read more information on sharing protocols at https://plos.org/protocols?utm_medium=editorial-email&utm_source=authorletters&utm_campaign=protocols.

As the corresponding author, your ORCID iD is verified in the submission system and will appear in the published article. PLOS supports the use of ORCID, and we encourage all coauthors to register for an ORCID iD and use it as well. Please encourage your coauthors to verify their ORCID iD within the submission system before final acceptance, as unverified ORCID iDs will not appear in the published article. *Only*  the individual author can complete the verification step; PLOS staff *cannot*  verify ORCID iDs on behalf of authors.

We look forward to receiving your revised manuscript.

Kind regards,

Yoko Hasegawa

Academic Editor

PLOS One

Journal Requirements:

If the reviewer comments include a recommendation to cite specific previously published works, please review and evaluate these publications to determine whether they are relevant and should be cited. There is no requirement to cite these works unless the editor has indicated otherwise.

Please review your reference list to ensure that it is complete and correct. If you have cited papers that have been retracted, please include the rationale for doing so in the manuscript text, or remove these references and replace them with relevant current references. Any changes to the reference list should be mentioned in the rebuttal letter that accompanies your revised manuscript. If you need to cite a retracted article, indicate the article’s retracted status in the References list and also include a citation and full reference for the retraction notice.

**Additional Editor Comments:**

The authors have satisfactorily addressed the principal concerns, and the revised interpretation is appropriately cautious. One final clarification of the statistical methods is required before acceptance.

Please specify the residual covariance structure, estimation method, and method used to calculate denominator degrees of freedom in the linear mixed models. Clarify how repeated observations within each viewing session and across the two sessions were accounted for, and briefly report how model assumptions were assessed. If these checks identify any issues affecting inference, please revise the analyses and corresponding results accordingly.

[Note: HTML markup is below. Please do not edit.]

[NOTE: If reviewer comments were submitted as an attachment file, they will be attached to this email and accessible via the submission site. Please log into your account, locate the manuscript record, and check for the action link "View Attachments". If this link does not appear, there are no attachment files.]

To ensure your figures meet our technical requirements, please review our figure guidelines: https://journals.plos.org/plosone/s/figures

You may also use PLOS’s free figure tool, NAAS, to help you prepare publication quality figures: https://journals.plos.org/plosone/s/figures#loc-tools-for-figure-preparation.

NAAS will assess whether your figures meet our technical requirements by comparing each figure against our figure specifications.

# Author comment: Author response to Decision Letter 3

**DOI:** 10.1371/journal.pone.0362672.r007

26 Oct 2026

Additional Editor Comments:

Please specify the residual covariance structure, estimation method, and method used to calculate denominator degrees of freedom in the linear mixed models. Clarify how repeated observations within each viewing session and across the two sessions were accounted for, and briefly report how model assumptions were assessed. If these checks identify any issues affecting inference, please revise the analyses and corresponding results accordingly.

Response

Thank you for this comment. We have revised the Statistical analysis section to specify the model structures, estimation method, denominator degrees-of-freedom method, and diagnostic procedures.

All models included fixed effects for video condition, categorical measurement time, and their interaction. Participant-specific random intercepts were included to account for dependence between observations from the same participant across the two viewing sessions. Additional session-specific random intercepts nested within participants were included for HR, LF/HF, HF, cortisol, amylase, and CgA. For LF and VLF, the session-specific random intercepts were omitted after initial fits yielded zero session-level variance estimates and estimation warnings.

Within each participant–session combination, a heterogeneous first-order autoregressive [ARH(1)] residual covariance structure was specified for HR and the HRV indices. This structure allows time-specific residual variances and correlations that depend on the separation between measurement times. A diagonal residual covariance structure allowing time-specific residual variances was specified for the salivary markers; dependence within and across sessions was additionally represented by the session-specific and participant-specific random intercepts, respectively. All models were fitted using restricted maximum likelihood, with denominator degrees of freedom calculated using the Satterthwaite approximation.

We checked convergence and covariance parameter estimates and visually examined residual histograms, Q–Q plots, residual-versus-fitted plots, and residual distributions by measurement time and video condition. These checks identified departures from normality and several large residuals for some outcomes. The corresponding observations were verified and retained, and the analyses were performed on the original measurement scale. We have explicitly acknowledged in the Limitations that the robustness of the model-based estimates, confidence intervals, and significance tests to these departures was not established.

Following this re-examination, we updated the fixed-effect results in Table 3, the estimated condition differences and confidence intervals in Table 4, and the between-condition differences in post-minus-pre changes in S3 Table. The Abstract, Results, Discussion, and Conclusions have been revised consistently with these results. In particular, the revised manuscript now reports a significant condition × time interaction for HR, but not for the other physiological indicators, and a significant average condition difference only for LF. Non-significant findings are not interpreted as evidence of equivalent responses between conditions.

Revised in: Abstract (pp. 2–3, lines 37–41), Materials and Methods (p10, lines 173-192); Results (pp. 17–18, lines 268–297); Table 3 (pp14-15), Table 4 (p16), Discussion (pp19-20, lines 317-337, p23 lines 407-410); Limitations of the Study (p26, lines 465-477), Conclusions (p27, lines 499-504) and Supporting Information (S3 Table).

Attachment Submitted filename: Response to Reviewers 20261024.docx

# Editor report: Decision Letter 3

**DOI:** 10.1371/journal.pone.0362672.r008

**Authors:** Yoko Hasegawa

29 Oct 2026

Cognitive and physiological responses to prolonged video viewing: Activities of the autonomic nervous system and hypothalamo-pituitary-adrenal axis in relation to video interest

PONE-D-25-64004R3

Dear Dr. Matsui,

We’re pleased to inform you that your manuscript has been judged scientifically suitable for publication and will be formally accepted for publication once it meets all outstanding technical requirements.

Within one week, you’ll receive an e-mail detailing the required amendments. When these have been addressed, you’ll receive a formal acceptance letter and your manuscript will be scheduled for publication.

An invoice will be generated when your article is formally accepted. Please note, if your institution has a publishing partnership with PLOS and your article meets the relevant criteria, all or part of your publication costs will be covered. Please make sure your user information is up-to-date by logging into Editorial Manager at Editorial Manager® and clicking the ‘Update My Information' link at the top of the page. For questions related to billing, please contact billing support.

If your institution or institutions have a press office, please notify them about your upcoming paper to help maximize its impact. If they’ll be preparing press materials, please inform our press team as soon as possible -- no later than 48 hours after receiving the formal acceptance. Your manuscript will remain under strict press embargo until 2 pm Eastern Time on the date of publication. For more information, please contact onepress@plos.org.

Kind regards,

Yoko Hasegawa

Academic Editor

PLOS One

Additional Editor Comments (optional):

Reviewers' comments:

# Editor report: Acceptance letter

**DOI:** 10.1371/journal.pone.0362672.r009

**Authors:** Yoko Hasegawa

PONE-D-25-64004R3

PLOS One

Dear Dr. Matsui,

I'm pleased to inform you that your manuscript has been deemed suitable for publication in PLOS One. Congratulations! Your manuscript is now being handed over to our production team.

At this stage, our production department will prepare your paper for publication. This includes ensuring the following:

* All references, tables, and figures are properly cited

* All relevant supporting information is included in the manuscript submission,

* There are no issues that prevent the paper from being properly typeset

You will receive further instructions from the production team, including instructions on how to review your proof when it is ready. Please keep in mind that we are working through a large volume of accepted articles, so please give us a few days to review your paper and let you know the next and final steps.

Lastly, if your institution or institutions have a press office, please let them know about your upcoming paper now to help maximize its impact. If they'll be preparing press materials, please inform our press team within the next 48 hours. Your manuscript will remain under strict press embargo until 2 pm Eastern Time on the date of publication. For more information, please contact onepress@plos.org.

You will receive an invoice from PLOS for your publication fee after your manuscript has reached the completed accept phase. If you receive an email requesting payment before acceptance or for any other service, this may be a phishing scheme. Learn how to identify phishing emails and protect your accounts at https://explore.plos.org/phishing.

If we can help with anything else, please email us at customercare@plos.org.

Thank you for submitting your work to PLOS One and supporting open access.

Kind regards,

PLOS One Editorial Office Staff

on behalf of

Dr. Yoko Hasegawa

Academic Editor

PLOS One
